# Cytokine-induced killer cells efficiently kill stem-like cancer cells of nasopharyngeal carcinoma via the NKG2D-ligands recognition

**DOI:** 10.18632/oncotarget.5280

**Published:** 2015-09-16

**Authors:** Fang Wei, Xiao-Xiang Rong, Rao-Ying Xie, Li-Ting Jia, Hui-Yan Wang, Yu-Juan Qin, Lin Chen, Hong-Fen Shen, Xiao-Lin Lin, Jie Yang, Sheng Yang, Wei-Chao Hao, Yan Chen, Sheng-Jun Xiao, Hui-Rong Zhou, Tao-Yan Lin, Yu-Shuang Chen, Yan Sun, Kai-Tai Yao, Dong Xiao

**Affiliations:** ^1^ Cancer Research Institute, Southern Medical University, Guangzhou 510515, China; ^2^ Institute of Comparative Medicine & Laboratory Animal Center, Southern Medical University, Guangzhou 510515, China; ^3^ Department of Oncology, Nanfang Hospital, Southern Medical University, Guangzhou 510515, China; ^4^ Children's Hospital Boston, Harvard Medical School, Boston, Massachusetts 02115, USA; ^5^ Department of Pathology, Guilin Medical College, Guilin 541001, China; ^6^ Guangzhou Digestive Disease Center, Guangzhou First People's Hospital, Guangzhou Medical University, Guangzhou 510180, China

**Keywords:** nasopharyngeal carcinoma, cytokine-induced killer cells, cancer stem cells, promoter-reporter gene strategy, time-lapse imaging

## Abstract

Cancer stem cells (CSCs) are considered to be the root cause for cancer treatment failure. Thus, there remains an urgent need for more potent and safer therapies against CSCs for curing cancer. In this study, the antitumor activity of cytokine-induced killer (CIK) cells against putative CSCs of nasopharyngeal carcinoma (NPC) was fully evaluated *in vitro* and *in vivo*. To visualize putative CSCs *in vitro* by fluorescence imaging, and image and quantify putative CSCs in tumor xenograft-bearing mice by *in vivo* bioluminescence imaging, NPC cells were engineered with CSC detector vector encoding GFP and luciferase (Luc) under control of Nanog promoter. Our study reported *in vitro* intense tumor-killing activity of CIK cells against putative CSCs of NPC, as revealed by percentage analysis of side population cells, tumorsphere formation assay and Nanog-promoter-GFP-Luc reporter gene strategy plus time-lapse recording. Additionally, time-lapse imaging firstly illustrated that GFP-labeled or PKH26-labeled putative CSCs or tumorspheres were usually attacked simultaneously by many CIK cells and finally killed by CIK cells, suggesting the necessity of achieving sufficient effector-to-target ratios. We firstly confirmed that NKG2D blockade by anti-NKG2D antibody significantly but partially abrogated CIK cell-mediated cytolysis against putative CSCs. More importantly, intravenous infusion of CIK cells significantly delayed tumor growth in NOD/SCID mice, accompanied by a remarkable reduction in putative CSC number monitored by whole-body bioluminescence imaging. Taken together, our findings suggest that CIK cells demonstrate the intense tumor-killing activity against putative CSCs of NPC, at least in part, by NKG2D-ligands recognition. These results indicate that CIK cell-based therapeutic strategy against CSCs presents a promising and safe approach for cancer treatment.

## INTRODUCTION

Nasopharyngeal carcinoma (NPC), one of the most common malignant tumors in Southeast Asia [1, 2], often invades adjacent regions and metastasizes to regional lymph nodes and distant organs, and the great potentiality of distant metastases remains the obstacles for survival improvement [3]. Novel and effective therapy for NPC is urgently warranted.

The development of cancer immunotherapy has received considerable attention in the last several decades [4–8]. Cytokine-induced killer (CIK) cells have demonstrated cancer-killing properties *in vitro*, killing an array of various cancer cell types and anti-tumor efficacy in mice and man [4, 7–9]. More importantly, CIK cells are capable of migrating to the tumor tissue, recognizing the abnormal vasculature and the tumor cells, and carrying out subsequent killing of tumor cells [8]. In the field of NPC, patients who received autologous CIK cell transfusion in combination with gemcitabine plus cisplatin chemotherapy had a higher overall survival and progression-free survival rates than patients with gemcitabine plus cisplatin chemotherapy [10]. However, intensive research work will still be required to improve CIK cell-based cancer therapy [8, 9].

Cancer stem cells (CSCs)/tumor-initiating cells (TICs), which are responsible for tumor initiation, maintenance, relapse and metastasis, and therapeutic resistance to conventional radio and chemotherapy, are considered to be the root cause for cancer treatment failure [11–19]. Therefore, there is an urgent need for more potent and safer therapies against CSCs for curing cancer.

Immune targeting of CSCs presents a promising and safe approach for cancer treatment, and one of the major advantages of most immunotherapeutic strategies is low or acceptable toxicity [20]. The previous report showed CIK cell-based therapy as an enhanced immune cell therapy in mice that can target stem-like lymphoma cells [21]. Cancer patient-derived CIK cells killed putative CSCs of autologous metastatic melanoma [22], and autologous metastatic bone sarcoma and soft-tissue sarcomas [23], which will be still required to be confirmed by further evidence (i.e., tumor sphere formation, time-lapse imaging, *in vivo* experiment, etc) and in various cancers. Additionally, so far, the antitumor activity of CIK cells against CSCs of NPC is completely unexplored. Against this background, in this study, we fully investigated the effects of CIK cell treatment on stem cell-like populations in NPC as well as the underlying mechanisms by using various methods.

## RESULTS

### CIK cell treatment resulted in the reduced stem cell-like properties of NPC cells

**Figure 1:**
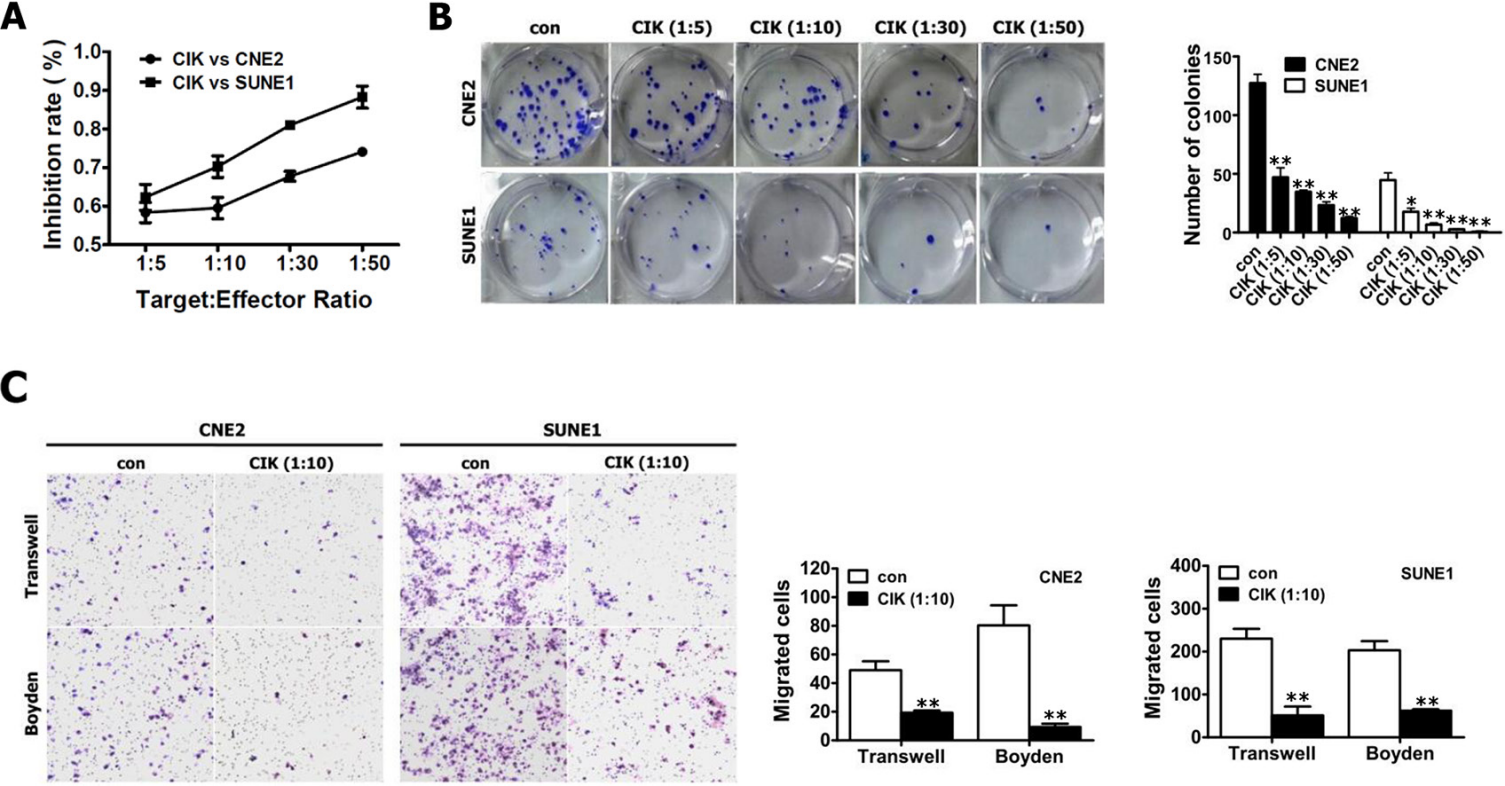
Evaluation of the *in vitro* cytotoxicity of CIK cells **A.** The proliferation ability of CNE2 and SUNE1 cells treated with CIK cells were analyzed by CCK8 assay. **B.** Colony formation assay for CNE2 and SUNE1 cells treated with CIK cells. **C.** NPC cells treated with CIK cells exhibited the reduced motility and invasion. The motility and invasion of CNE2 and SUNE1 cells were analyzed with an *in vitro* migration assay using a transwell chamber and an *in vitro* invasion assay using a matrigel-coated Boyden chamber, respectively. The migrated cells were plotted as the average number of cells per field of view from 3 different experiments, as described in the materials and methods section.

Flow cytometric analysis of CIK cell phenotype was illustrated in “Supplemental Results” section and Fig. S1. Since our results from “Supplemental Results” section showed that CIK cells demonstrated a strong cancer killing activity (CKA) *in vitro* against NPC cells (Fig. 1), we further explore the effects of CIK cell treatment on stem cell-like populations in NPC.

**Figure 2:**
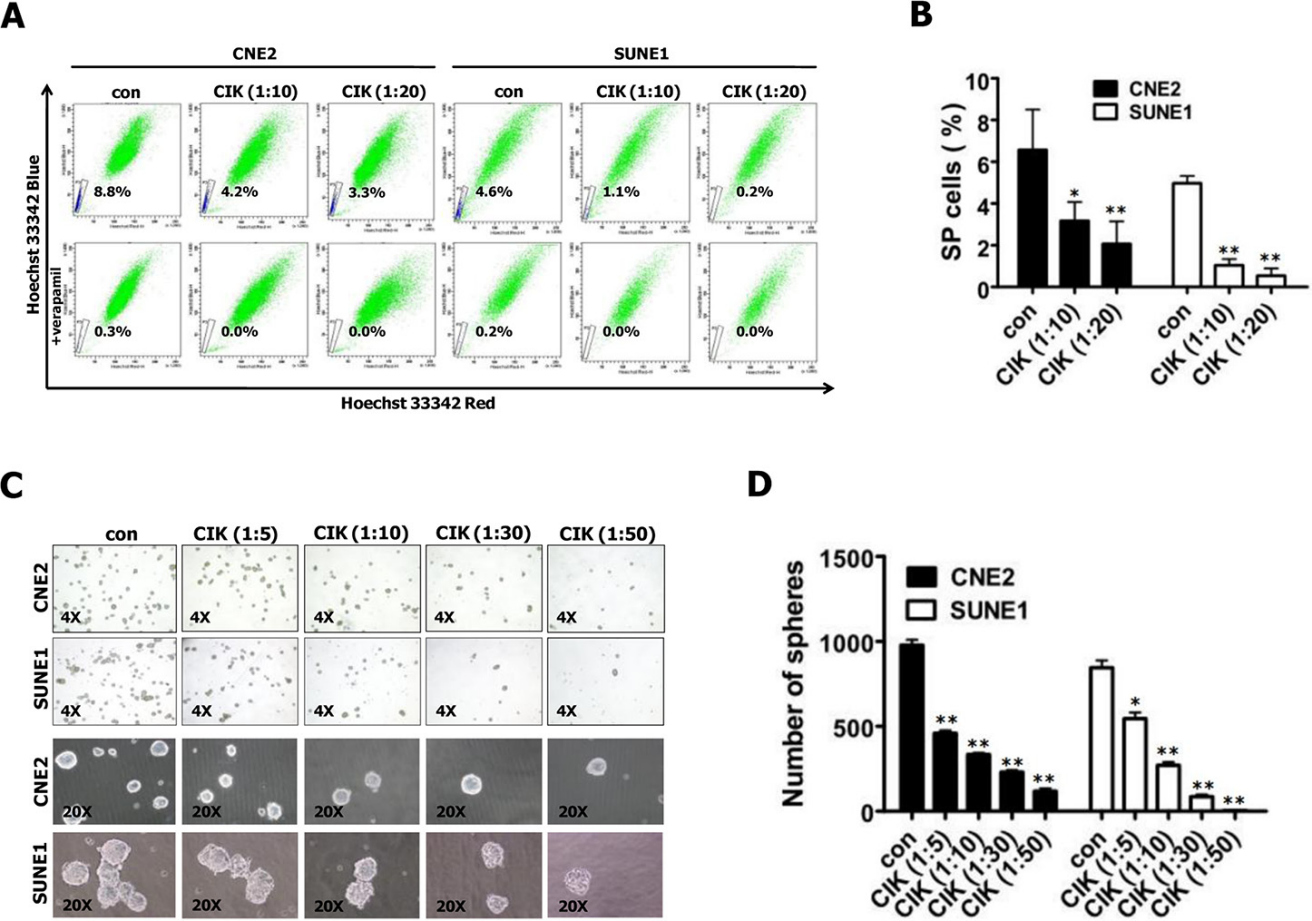
CIK cells were active against stem-like cancer cells of NPC **A–B.** CIK cell treatment led to the decreased size of SP cells. SP cell profiles in the presence of verapamil are shown in the bottom panels. The percentages of SP cells are indicated. Data represent the mean ± SD; *n* = 3. **C–D.** Images showing tumor sphere formation in CIK-treated NPC cells. Sphere size and density are shown in the left panels (C), and the number of spheres is shown in the right panels (D).

Sde populations (SPs) among NPC cells and tumorspheres have been reported to exhibit CSC characteristics [24–27]. We first tested the effects of CIK cell treatment on the percentages of SP cells in CNE2 and SUNE1 cells, and found that CIK cell treatment dramatically decreased the percentage of SP cells in CNE2 and SUNE1 cells (Fig. 2A). This data was confirmed on several occasions, and found to be statistically significant (Fig. 2B). Taken together, our results demonstrate that CIK cell treatment can remarkably reduce the cancer stem cell-like SPs in NPC cells.

We further examined the ability of CNE2 and SUNE1 cells to form tumor spheres after treated with CIK cells at different E:T ratios by tumorsphere formation assay. The results showed that CIK-treated CNE2 and SUNE1 cells demonstrated a dramatical decrease in tumorsphere formation efficiency in a dose-dependent manner (Fig. 2C, 2D). Together, our results indicate that CIK cells can efficiently kill cancer stem-like cell populations within NPC cell lines *in vitro*.

### Visualization of cancer stem-like cells in NPC with a “CSC detector”

**Figure 3:**
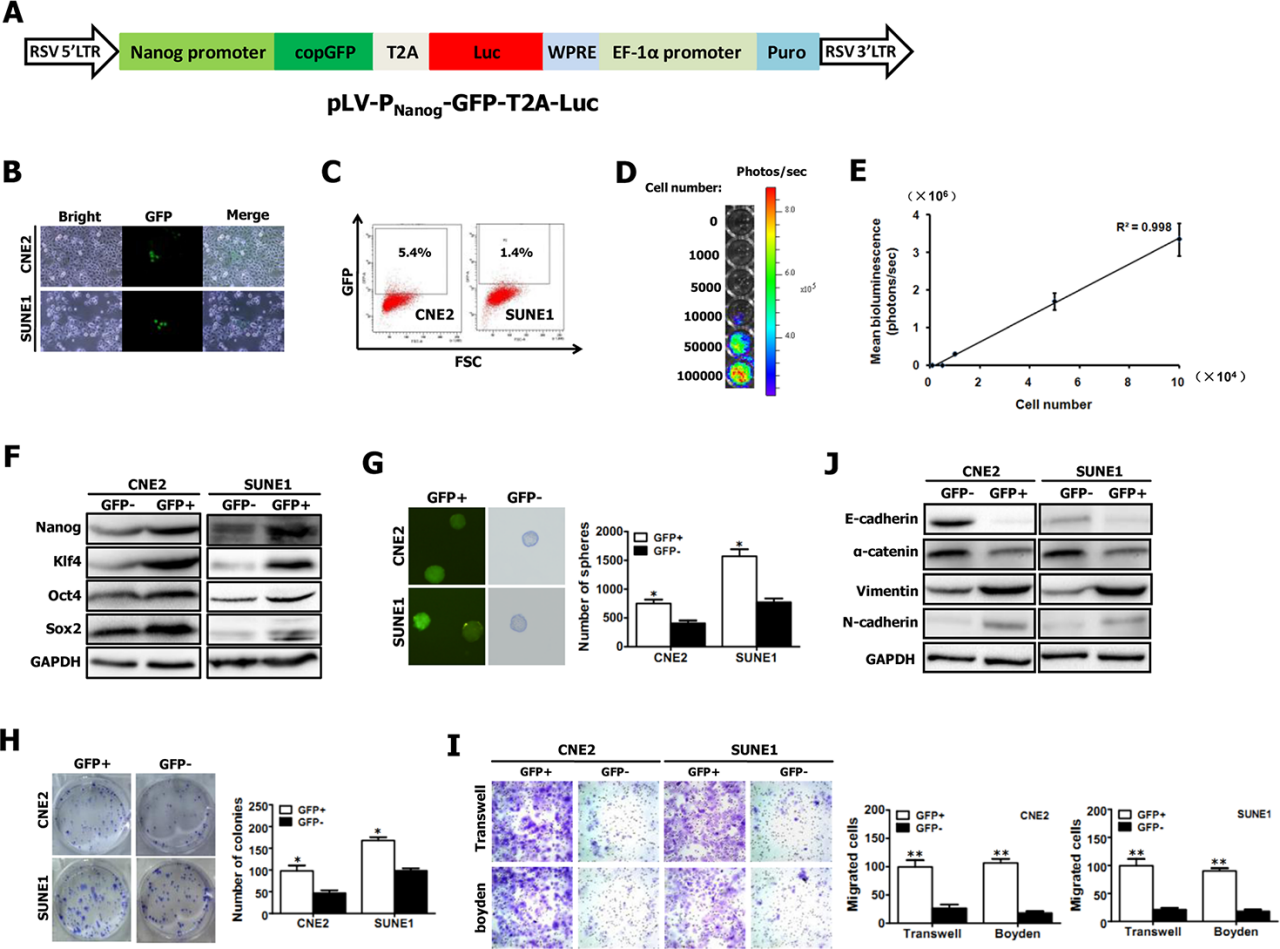
Visualization of putative CSCs of NPC with a “CSC detector” **A.** Schematic diagram of lentiviral vector pLV-P_Nanog_-GFP-T2A-Luc, in which GFP and Luc expression was controlled by human Nanog promoter. The construct map is not drawn to the scale. Abbreviations: **Luc**: firefly luciferase; **GFP**: green fluorescent protein. **B–C.** Representative GFP expression was measured in NPC cell lines (e.g., CNE2 and SUNE1) carrying P_Nanog_-GFP-T2A-Luc transgene by inverted fluorescence microscope (B) and by flow cytometry (C). **D.** CNE2 cells harboring Luc have robust reporter gene expression as shown by bioluminescence imaging (BLI). **E.** A strong correlation exists between BLI signal and CNE2 cell number. **F–G.** GFP+ and GFP- fractions sorted from CNE2 and SUNE1 cells carrying P_Nanog_-GFP-T2A-Luc transgene (shown in Fig. 3B, 3C) by fluorescence-activated cell sorting (FACS) were subjected to Western blotting for the detection of Nanog, Oct4, Sox2 and Klf4 expression (F), and tumor spheroid formation assay (G). **H–I.** The growth (H), migration (I) and invasion (I) of GFP+ and GFP- NPC cells were evaluated by colony formation assay, transwell migration assay and matrigel-coated Boyden chamber assay, respectively. GFP+ and GFP- fractions were sorted from CNE2 and SUNE1 cells carrying P_Nanog_-GFP-T2A-Luc transgene (shown in Fig. 3B, 3C) by FACS. **J.** GFP+ and GFP- fractions sorted from CNE2 and SUNE1 cells carrying P_Nanog_-GFP-T2A-Luc transgene (shown in Fig. 3B, 3C) by FACS were subjected to Western blotting for the detection of E-cadherin, α-catenin, vimentin and N-cadherin expression.

The commonly used optical molecular imaging techniques include fluorescence and bioluminescence imaging [28–30] which have their own advantages. Nanog has been frequently used as CSC-related markers to identify CSC population from clinical samples and cell lines in various cancers, including NPC [31–34]. Therefore, based on promoter-reporter gene strategy, we have devised and constructed the lentivirus vector of pLV-P_Nanog_-GFP-T2A-Luc harboring GFP and luciferase (Luc) under control of human Nanog promoter (Fig. 3A), which allows us to integrate respective advantages of fluorescence and bioluminescence imaging to visualize putative CSCs within *in vitro* cultured cancer cells and *in vivo* optically image and quantify a rare population of putative CSCs in human tumor xenograft-bearing mice.

To optically visualize putative CSCs, CNE2 and SUNE1 cells were infected with lentiviruses carrying P_Nanog_-GFP-T2A-Luc transgene (Fig. 3A). 10 days after infection, we found that GFP was highly expressed in a small percentage of stably infected CNE2 and SUNE1 cells (Fig. 3B). Fluorescence activated cell-sorting (FACS) analysis revealed the average GFP expression was 5.4% in CNE2 cells and 1.4% in SUNE1 cells (Fig. 3C). Importantly, the detection of Luc expression showed a strong linear correlation (*r*^2^ = 0.998) between the total CNE2 cell numbers and the bioluminescence signals (Fig. 3D, 3E).

Next, GFP-positive (GFP+) and GFP-negative (GFP-) cells were sorted, and then gene expression was analyzed and the respective assays mentioned below were performed (Fig. 3F–3J). Fig. 3F showed that the significantly increased levels of the known CSC-related genes (i.e., Nanog, Oct4, Sox2 and Klf4) were observed in sorted GFP+ cells, as compared with GFP- cells, indicating that GFP+ cells might have stem-cell–like characteristics.

CSCs can form tumorspheres *in vitro* in a non-attached culture condition [25, 35]. The increased sphere forming ability of GFP+ cells was confirmed (Fig. 3G). Moreover, approximately 8.3% GFP+ cells (for CNE2) and 17.5% GFP+ cells (for SUNE1) can form spheres and all spheres exhibited GFP expression (Fig. 3G). Additionally, colony formation assay showed that GFP+ cells were able to induce more colonies than GFP- cells (Fig. 3H). Together, GFP+ cancer cells exhibit the characteristics of CSCs.

Furthermore, our data revealed that GFP+ cells illustrated a dramatical increase in motile capacity by 2.7 folds (for CNE2) and 3.8 folds (for SUNE1), and a remarkable increase in invasive ability by 4.9 times (for CNE2) and 4 times (for SUNE1), compared with GFP- cells (Fig. 3I). Epithelial-mesenchymal transition (EMT) has been shown to endow cancer cells with strong invasive ability. Thus we examined the molecular markers of EMT in both GFP+ and GFP- cells by Western blot. As expected, GFP+ cells exhibited the molecular characterization of mesenchyma with reduced expression of E-cadherin and α-catenin, and enhanced expression of vimentin and N-cadherin (Fig. 3J). These data indicate that the increased migratory and invasive capacity of GFP+ cancer cells is coincident with EMT phenotype.

Our aforementioned data demonstrate that GFP+ cancer cells harboring P_Nanog_-GFP-T2A-Luc transgene exhibit the characteristics of CSCs. Therefore, the newly established NPC cell lines harboring P_Nanog_-GFP-T2A-Luc transgene allows us to *in vitro* visualize putative CSCs by fluorescence imaging and *in vivo* optically image and quantify putative CSCs in human tumor xenograft-bearing mice by bioluminescence imaging.

### Tumor-killing activity of CIK cells remained equally effective against both putative CSCs and non-CSCs of NPC

**Figure 4:**
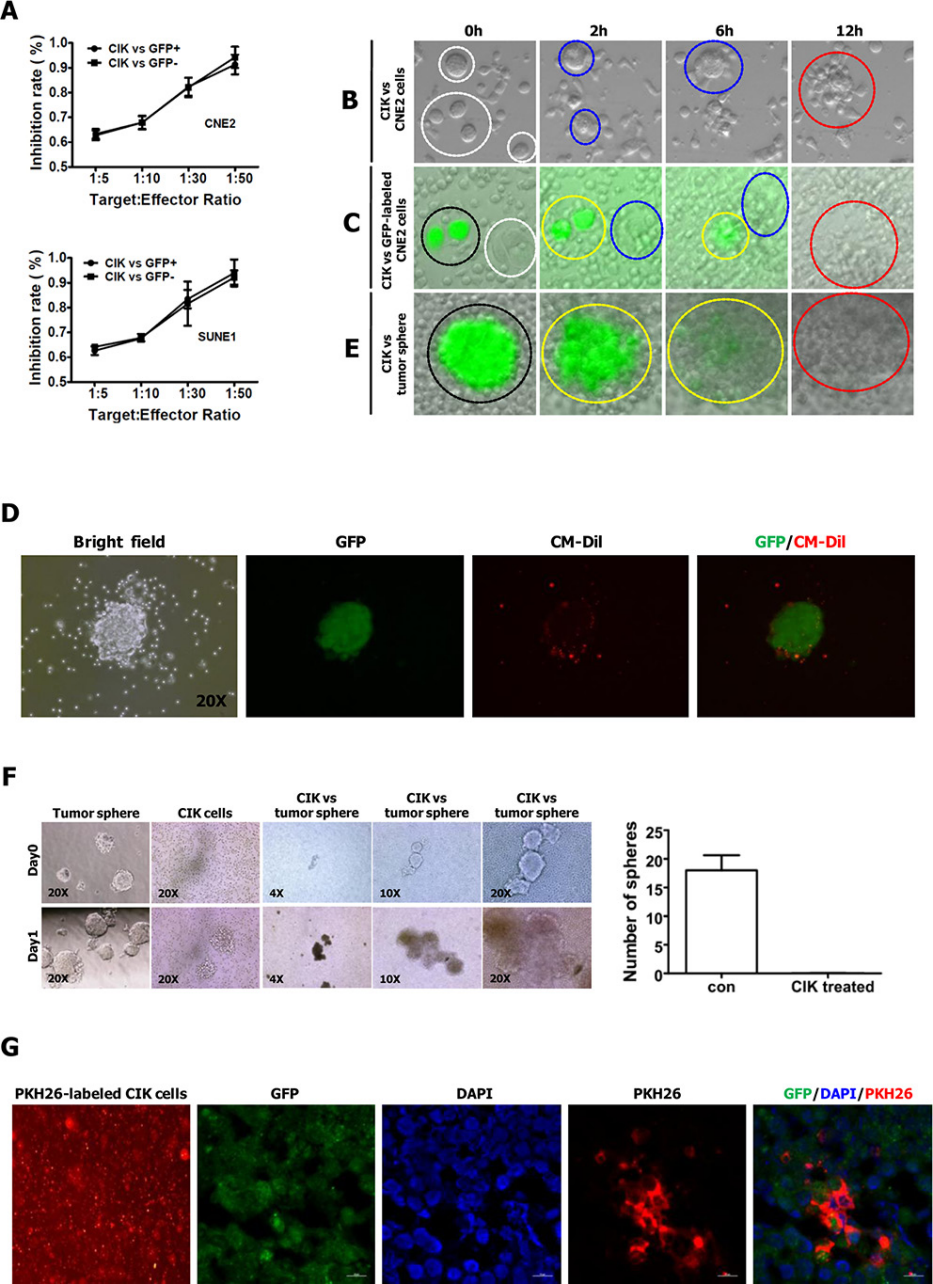
CIK cells have direct killing effect on stem-like cancer cells of NPC **A.** The antitumor activity of CIK cells was equally intense against GFP+ and GFP- NPC cells. **B.** Time-lapse imaging (see supplemental Movie 1) used to observe the interactions between CNE2 cells and CIK cells. **C.** Time-lapse imaging (see supplemental Movie 2) used to observe the interactions between CNE2 cells (harboring P_Nanog_-GFP-T2A-Luc transgene) and CIK cells. **D.** Representative pictures of GFP+ tumor sphere surrounded by CM-Dil-labeled CIK cells. GFP+ tumor sphere was generated from GFP+ cells sorted from CNE2 cells harboring P_Nanog_-GFP-T2A-Luc transgene. Red fluorescent dye CM-Dil was used to label CIK cells. **E.** Time-lapse imaging (see supplemental Movie 5) used to observe the interactions between GFP+ tumor sphere (derived from GFP+ cells sorted from CNE2 cells harboring P_Nanog_-GFP-T2A-Luc transgene) and CIK cells. The explanations of the Figure 4B, 4C, 4E are presented in figure legends of Supplemental Movies. **F.** CIK cells efficiently killed tumor spheres derived from CNE2 cells. Representative pictures of sphere are shown in the left panels, and the number of spheres is shown in the right panels. **G.** Infiltration of PKH26-labeled CIK cells surrounding GFP+ stem-like cancer cells was shown by red fluorescent at frozen tissue section. GFP+ cells sorted from CNE2 cells harboring P_Nanog_-GFP-T2A-Luc transgene were subcutaneously injected into the dorsal thigh of NOD/SCID mice. After implanted tumor formed, CIK cells labeled with red fluorescent dye PKH26 were injected intravenously once a day. 3 days after PKH26-labeled CIK cell implantation, mice were sacrificed, and tumors were dissected, followed by frozen section.

On the basis of GFP expression, NPC cells harboring P_Nanog_-GFP-T2A-Luc transgene were sorted into GFP+ and GFP- fractions that served as targets to evaluate separately the antitumor activity of CIK cells against GFP+ (putative CSCs) and GFP- cells. Our results showed that the tumor killing activity of CIK cells remained equally effective against both GFP+ and GFP- NPC cells (Fig. 4A). In our previous study, a stem cell-like subpopulation (PKH26+) has been successfully identified in NPC cell lines (e.g., CNE2 cells) using a label-retention technique [36]. In this study, stem cell-like subpopulation within CNE2 and SUNE1 cells were labeled by PKH26, as described previously [36]. PKH26+ and PKH26- fractions were sorted by FACS for subsequent analyses. Our findings showed CIK cells can equally efficiently kill both PKH26+ and PKH26- cells sorted from PKH26-labelled CNE2 or SUNE1 cells (Fig. S2A). Summarily, our data indicate that the antitumor activity of CIK cells was equally intense against putative CSCs and non-CSCs of NPC.

### Time-lapse imaging revealed the direct killing effect of CIK cells on stem-like cancer cells of NPC

To optically visualize the killing process of CIK cells against cancer cells, putative CSCs or tumor spheres, we performed time-lapse video recording (Fig. 4B, 4C, 4E, and supplemental Movies 1–5). Firstly, we visualized the killing process of CIK cells against CNE2 cells by time-lapse imaging, and found that one cancer cell was generally attacked simultaneously by many CIK cells and finally killed by CIK cells (Fig. 4B and supplemental Movie 1).

Secondly, we used time-lapse video recording to visualize the killing process of CIK cells against putative CSCs and non-CSCs of NPC (Fig. 4C, Fig. S2B, S2C and supplemental Movies 2–4). As mentioned above, GFP+ CNE2 cells harboring P_Nanog_-GFP-T2A-Luc transgene exhibit the characteristics of CSCs, and the slow-cycling PKH26+ cells within NPC cells are enriched for CSCs [36]. Time-lapse video microscopy optically illustrated that the tumor killing activity of CIK cells remained equally effective against both GFP+ (i.e., putative CSCs) and GFP- cells (Fig. 4C, and supplemental Movie 2), and both PKH26+ (i.e., putative CSCs) and PKH26- cells (Fig. S2B, S2C and supplemental Movies 3–4), indicating that the antitumor activity of CIK cells was equally intense against putative CSCs and non-CSCs of NPC. Furthermore, time-lapse imaging illustrated that each of GFP+ or GFP- cells, and PKH26+ or PKH26- cells was usually attacked simultaneously by many CIK cells and finally killed by CIK cells (supplemental Movies 1–4).

As tumor-derived spheres are highly enriched in CSCs [24, 25], we further employed time-lapse fluorescence imaging to visualize the killing process of CIK cells against tumor sphere of NPC. As shown in Fig. 4D, GFP+ tumor sphere was surrounded by many red fluorescence-labeled CIK cells when CM-Dil-labeled CIK cells were added into culture medium with GFP+ tumor spheres. Time-lapse fluorescence video microscopy fully revealed that one tumor sphere exhibiting green fluorescence from GFP was attacked simultaneously by many CIK cells (Fig. 4E and supplemental Movie 5). During the process of the continuous attack and killing of CIK cells, the GFP+ tumor sphere gradually became smaller and it's shape became irregular, until cancer cells within GFP+ tumor sphere were killed completely, while green fluorescence emitted from cancer cells became more and more weak, until it became nearly or completely undetectable at the indicated time range (Fig. 4E and supplemental Movie 5). Moreover, complete or near-complete loss of fluorescence in the most right picture of Fig. 4E demonstrated that cancer cells within GFP+ tumor sphere in the most left picture of Fig. 4E had been scavenged by CIK cells. Furthermore, tumor spheres were efficiently scavenged by CIK cells when CIK cells were co-cultured with tumor spheres formed from CNE2 cells for one day (Fig. 4F). Further *in vivo* analysis established that red fluorescence-labeled CIK cells could infiltrate tumors formed from GFP+ cells sorted from CNE2 cells harboring P_Nanog_-GFP-T2A-Luc transgene (Fig. 4G). Together, these findings strongly suggest that CIK cells are active against putative CSCs of NPC.

### CIK cells killed stem-like cancer cells of NPC via NKG2D-ligands recognition

The MHC-unrestricted tumor-killing activity of CIK cells is mainly based on the interaction between their membrane receptor NKG2D molecules on CIK cells and MIC A/B or ULBPs molecules on tumor cells [9, 37, 38]. Our aforementioned results revealed that tumor killing activity of CIK cells remained equally effective against both putative CSCs and non-CSCs of NPC. Therefore, the anti-NKG2D antibody blocking assay was employed to define whether CIK cells might kill putative CSCs of NPC by NKG2D-ligands recognition.

**Figure 5:**
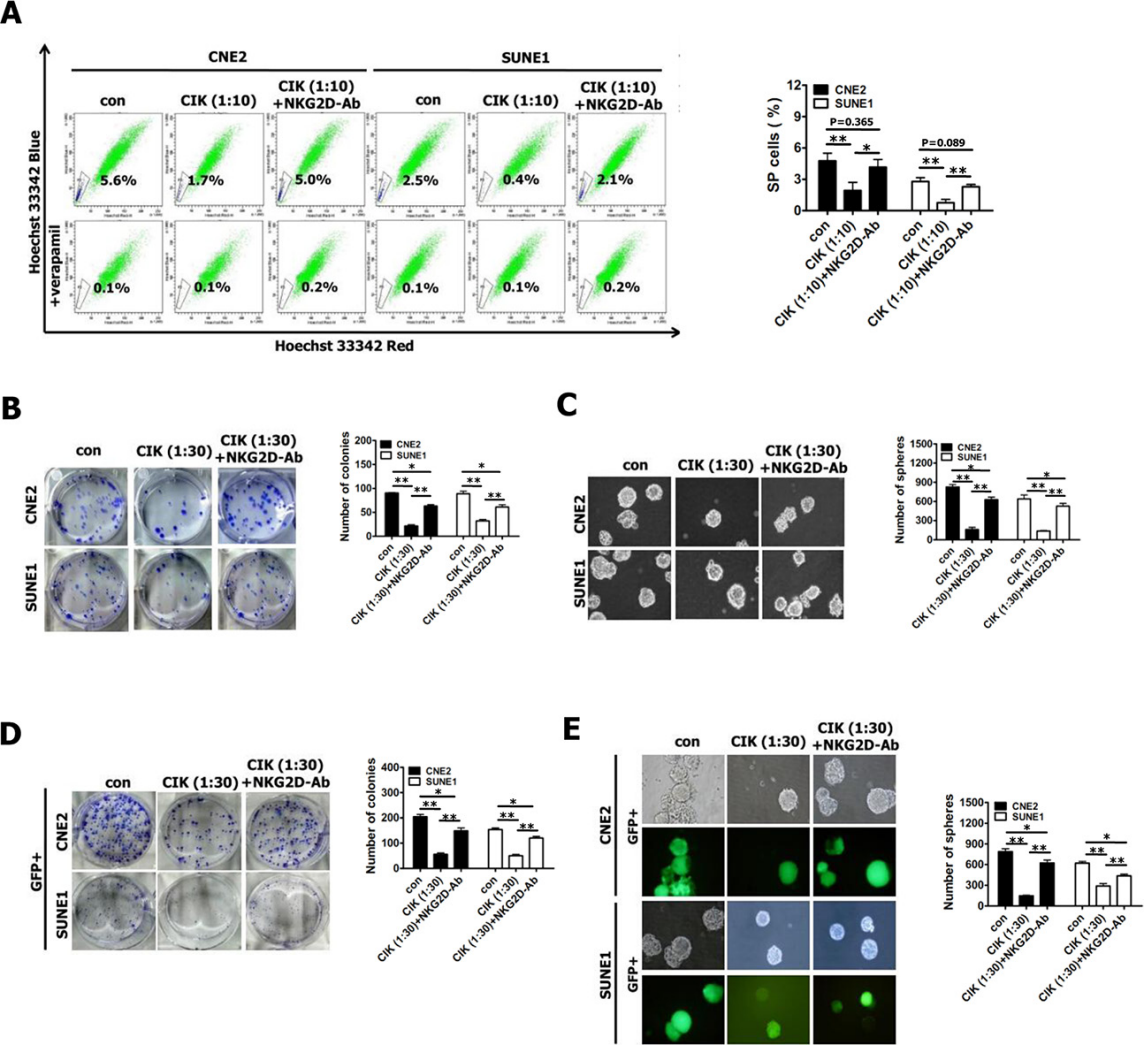
Blocking of NKG2D with antibody significantly reduced tumor killing activity of CIK cells against putative CSCs **A–C.** The percentages of SP cells (A), colony formation assay (B) and tumor sphere formation assay (C) in CNE2 and SUNE1 cells treated with CIK cells alone and CIK cells which were pre-incubated with anti-NKG2D antibody for 30 min. **D–E.** Colony formation assay (D) and tumor sphere formation assay (E) of GFP+ cells (sorted from CNE2 and SUNE1 cells harboring P_Nanog_-GFP-T2A-Luc transgene) treated with CIK cells alone and CIK cells which were pre-incubated with anti-NKG2D antibody for 30 min.

We first examined the effects of CIK cell treatment plus anti-NKG2D neutralizing antibody on the percentages of SP cells in CNE2 and SUNE1 cells. We found that after CIK cell treatment alone, the size of SP cells in CNE2 and SUNE1 cells significantly decreased from 4.77 ± 0.74% and 2.80 ± 0.36% (before CIK cell treatment) to 1.93 ± 0.78% and 0.77 ± 0.32% (after CIK cell treatment) (Fig. 5A), respectively, whereas addition of anti-NKG2D antibody mostly rescued the reduced SP cell percentages in CIK-treated CNE2 and SUNE1 cells from 1.93 ± 0.78% and 0.77 ± 0.32% to 4.17 ± 0.74% and 2.30 ± 0.20% (Fig. 5A), respectively. Together, NKG2D blockade mostly restores CIK treatment-induced decrease in the percentages of SP cells within NPC cells.

Subsequently, our results showed that addition of anti-NKG2D antibody partially but significantly rescued CIK treatment-induced decrease in colony formation ability (Fig. 5B) and tumor sphere formation ability (Fig. 5C) of CIK-treated CNE2 and SUNE1 cells, suggesting that NKG2D blockade partially inhibited tumor-killing activity of CIK cells.

Finally, we employed GFP+ putative CSCs sorted from CNE2 and SUNE1 cells harboring P_Nanog_-GFP-T2A-Luc transgene to examine whether addition of anti-NKG2D antibody could block the direct killing effect of CIK cells against putative CSCs of NPC by colony formation assay and tumor sphere formation assay. We observed that addition of anti-NKG2D antibody significantly but partially restored CIK treatment-induced remarkable reduction in colony formation ability (Fig. 5D) and tumor sphere formation ability (Fig. 5E) of CIK-treated GFP+ putative CSCs of CNE2 and SUNE1 cells, indicating that NKG2D blockade partially blocked the direct tumor-killing activity of CIK cells against putative CSCs of NPC.

All these results certainly indicate that the direct tumor-killing effect of CIK cells against putative CSCs of NPC, at least in part, by NKG2D-ligands recognition.

### Pro-inflammatory cytokine secretion by CIK cells into co-culture media

**Figure 6:**
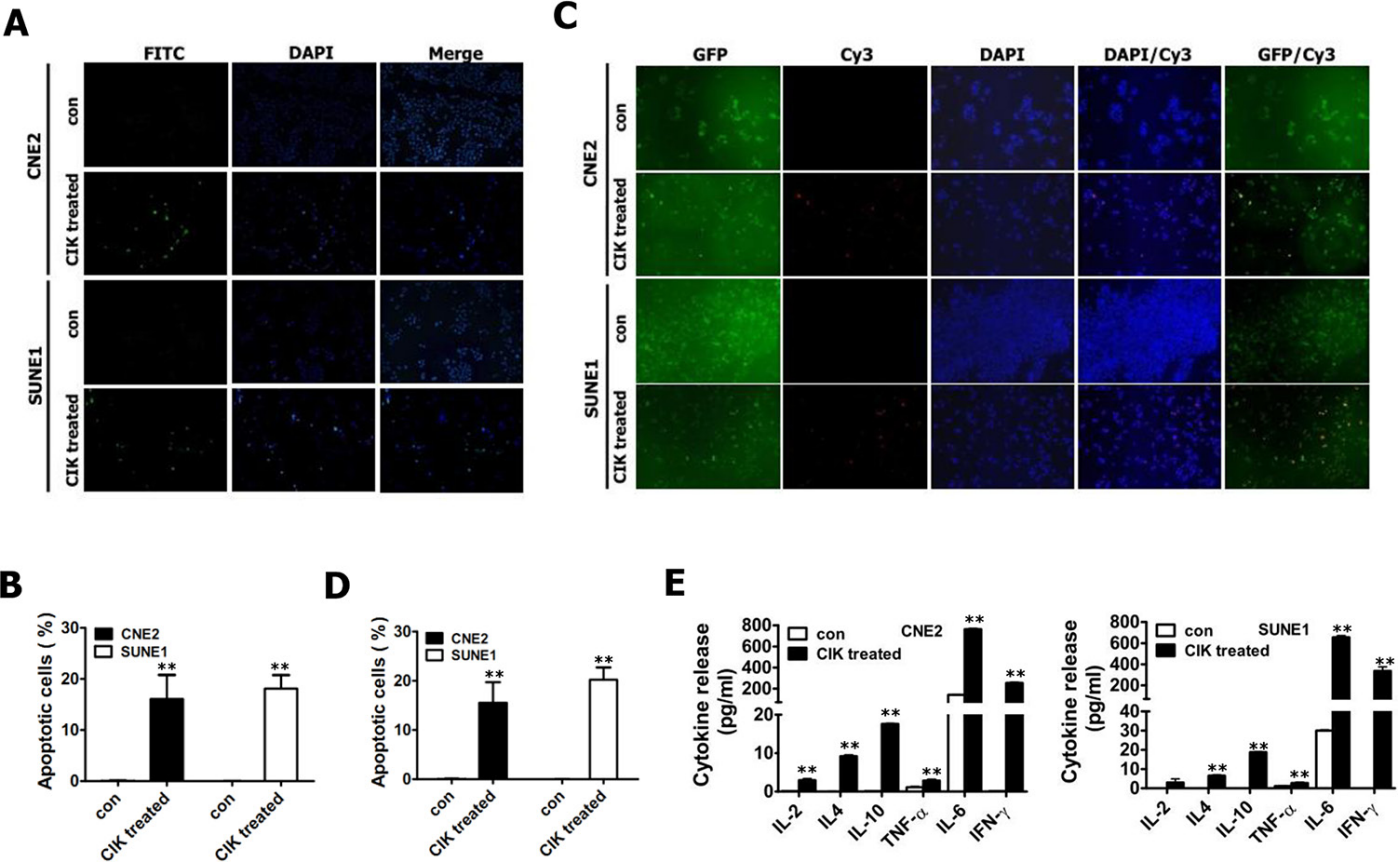
CIK cells efficiently killed NPC cells (including putative CSCs) through the induction of apoptosis and secretion of immune cytokines **A–B.** CIK-treated CNE2 and SUNE1 cells exhibited significantly increased apoptotic cells detected by TUNEL staining. **C–D.** CIK-treated GFP+ CNE2 and SUNE1 cells showed significantly enhanced apoptotic cells assayed by TUNEL staining. After CNE2 and SUNE1 cells (A-B) or GFP+ fractions sorted from CNE2 and SUNE1 cells carrying P_Nanog_-GFP-T2A-Luc transgene by FACS (C-D) were co-cultured with CIK cells for 24 h, cancer cells were washed three or four times with PBS to fully remove suspended CIK cells, followed by TUNEL staining to detect cells undergoing apoptosis. The FITC-conjugated green signal (A) and Cy3-conjugated red signal (C) showed the presence of TUNEL-positive cells. DAPI (blue) is used as nuclear counterstain. **E.** The concentrations of IL-2, IL-4, IL-6, IL-10, TNF-α and IFN-γ in 24 h co-culture supernatant were detected by ELISA.

Although the non-MHC-restricted tumor-killing activity of CIK cells is mainly mediated by the interaction of NKG2D with stress-inducible molecules (i.e., MIC A/B and ULBPs) on target cells [39–41], the pro-inflammatory cytokines secreted by CIK cells also play a role in killing cancer cells. Thus we also assessed the cytokine protein production by CIK cells. NPC cells were co-cultured with CIK cells at an E:T ratio of 30:1 for 24 h, and the co-culture supernatants were then harvested and assayed for levels of IL-2, IL-4, IL-6, IL-10, TNF-α and IFN-γ by ELISA. ELISA showed that CIK cells produced significant amounts of IFN-γ and IL-6 (Fig. 6E); whereas slight amounts of IL-2, IL-4, IL-10 and TNF-α were produced by CIK cells (Fig. 6E), suggesting that compared with IL-2, IL-4, IL-10 and TNF-α, IFN-γ and IL-6 might play an important role in tumor-killing activity of CIK cells against NPC cells.

### CIK-treated GFP+ NPC cells exhibited significantly increased apoptotic cells

Our findings illustrated that CIK cells efficiently killed cancer cells at least by both NKG2D-ligands recognition (Fig. 5) and effector cytokines secreted by CIK cells (Fig. 6E). Next, TUNEL staining was used to detect the apoptosis of putative CSCs and non-CSCs of NPC induced by CIK cells. TUNEL staining revealed about 16.06 ± 4.71% and 18.11 ± 2.66% of apoptotic cells in CNE2 and SUNE1 cells treated with CIK cells (Fig. 6A, 6B). Moreover, GFP+ putative CSCs of NPC were also treated for 24 h with CIK cells, and subsequently TUNEL analysis demonstrated that GFP+ putative CSCs showed a markedly high apoptotic rate compared to control (Fig. 6C, 6D). Summarily, our studies suggest that CIK cells efficiently kill putative CSCs and non-CSCs of NPC by inducing apoptosis.

### *In vivo* bioluminescence imaging of tumor-killing activity of CIK cells against putative CSCs of NPC

Our aforementioned *in vitro* findings fully revealed that CIK cells had a strong ability to attack and efficiently kill both putative CSCs and non-CSCs of NPC, as shown by various efficient *in vitro* approaches. We next evaluated the tumor-killing activity of CIK cells *in vivo* against putative CSCs of NPC in NOD/SCID mice by *in vivo* optical imaging and other methods.

**Figure 7:**
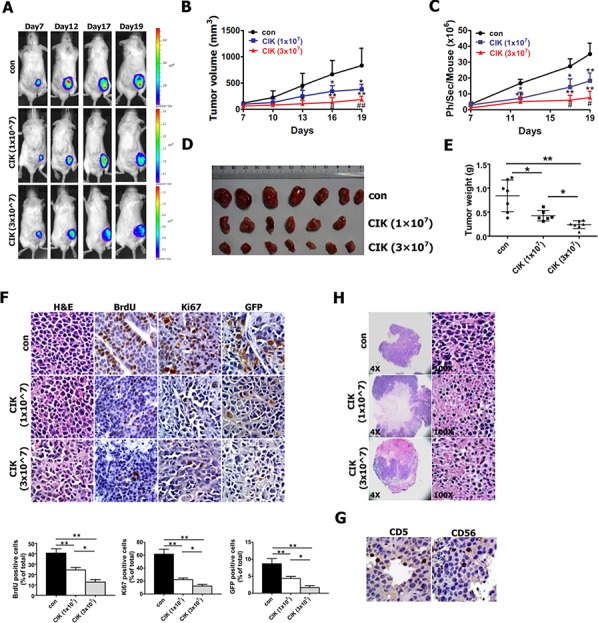
*In vivo* activity of CIK cells against CNE2 cells harboring GFP-T2A-Luc in NOD/SCID mice As mentioned in Materials and methods section, NOD/SCID mice were subcutaneously implanted with 1 × 10^6^ CNE2 cells harboring P_Nanog_-GFP-T2A-Luc transgene. 7 days after tumor cell implantation, 1 × 10^7^ and 3 × 10^7^ CIK cells were infused by tail vein injection into recipient mice once every two days. **A.** Series of *in vivo* bioluminescence images (taken at the indicated times) of 3 representative NOD/SCID mice from CIK- (1 × 10^7^), CIK- (3 × 10^7^) and vehicle-treated groups before and after CIK cell treatment. **B.** Growth curve of tumor volumes. **C.** Quantification analysis of bioluminescence signal of tumor-bearing mice treated with CIK cells (1 × 10^7^ and 3 × 10^7^) or with vehicle control. **D.** Representative picture of tumors formed. **E.** Tumors were weighted. **F.** BrdU, Ki67 and GFP-stained sections of transplanted tumors formed by CNE2 cells at 19 days after subcutaneous transplantation. The percentages of BrdU-, Ki67- or GFP-positive cancer cells were calculated by the total number of BrdU-, Ki67- or GFP-positive cells over total number of cancer cells. **G.** Infiltration of CIK cells at tumor sites was shown by immunohistochemistry (Ab anti-CD5 and Ab anti-CD56) at the end of experiment. **H.** A significant increase in transplanted tumor tissue necrosis and apoptosis was observed in CIK-treated mice compared with controls at the end of the experiment.

For *in vivo* bioluminescence imaging of putative CSCs, NOD/SCID mice were subcutaneously implanted with the newly established CNE2 cell line harboring P_Nanog_-GFP-T2A-Luc transgene. One week after CNE2 cell implantation, CIK cells were injected intravenously into tumor-bearing NOD/SCID mice once every two days at doses of 1 × 10^7^ and 3 × 10^7^ cells per mouse, followed by the indicated detections and analyses described below. Following CIK cell treatment, a significant reduction of tumor growth was observed in CIK-treated mice compared with untreated controls (Fig. 7A, 7B and Fig. S3), and as early as 6 days or 9 days after CIK cell treatment, the growth of transplanted tumors between CIK-treated mice and untreated controls or between two CIK-treated groups became statistically significant (Fig. 7B), respectively.

On day 19, all tumors were excised from NOD/SCID mice and weighed, which illustrated the strong anti-tumor effects of CIK cells against CNE2 cells (Fig. 7D, 7E), followed by histological and immunohistochemical analysis. CIK cells that were injected intravenously at doses of 1 × 10^7^ and 3 × 10^7^ cells per mouse inhibited tumor weight by 49% and 74%, respectively (Fig. 7E). Additionally, the control and CIK cell-injected NOD/SCID mice exhibited body weight gains of 121~128%, and no difference in body weight of NOD/SCID mice was found between the CIK–treated and the control mice, indicating that CIK cell therapy did not produce animal toxicity (data not shown). The results of immunohistochemical analysis revealed that the number of hyperproliferative BrdU- and Ki67-positive tumor cells in two CIK-treated groups were significantly decreased compared with control (Fig. 7F), while the percentages of BrdU- and Ki67-positive tumor cells between two CIK-treated groups became statistically significant (Fig. 7F).

Furthermore, we also assessed the antitumor activity of CIK cells against NPC cells in nude mouse xenograft assays (Fig. S4). Our results showed that CIK cell treatment inhibited tumor growth of NPC cells in nude mice (Fig. S4).

The above-mentioned data from these conventional methods fully exhibited that a significant delay in the growth of subcutaneous tumor xenografts was observed in CIK-treated NOD/SCID and nude mice (Fig. 7B, 7D, 7E, 7F and Fig. S4A-S4C). The above-established subcutaneous tumor xenografts, which expressed both GFP and Luc transgenes under the control of a stem cell-specific Nanog promoter, allow us to *in vivo* non-invasively optically image and quantify putative CSCs within tumor xenografts in NOD/SCID mice by noninvasively detecting Luc signal via using *in vivo* bioluminescence imaging. Therefore, when tumor sizes of subcutaneous tumors were determined by caliper measurement, tumor-bearing NOD/SCID mice were imaged at the indicated times by *in vivo* bioluminescence imaging to monitor the changes in the number of putative CSCs within tumor xenografts.

Series of *in vivo* bioluminescence images taken of 3 representative mice from each group are presented in Fig. 7A, while quantification of the bioluminescence signal generated from each tumor-bearing mouse is shown in Fig. 7C. As shown in Fig. 7A, 7C following CIK cell treatment, the significant reduction of bioluminescence signal was found in CIK-treated mice compared with untreated controls, and as early as 12 days or 17 days post-treatment of CIK cells, the intensities of bioluminescence signal between CIK-treated mice and untreated controls or between two CIK-treated groups became statistically significant, respectively, indicating that a general downward trend in the number of putative CSCs within tumor xenografts after CIK cell treatment can be readily, noninvasively and optically monitored by *in vivo* bioluminescence imaging.

Since the expression of both GFP and Luc transgenes in tumor xenografts is driven by the same Nanog promoter, we intended to further quantify number of putative CSCs by determining the percentage of GFP-positive cells in paraffin-embedded section of tumor xenografts through GFP antibody-based staining method. As shown in Fig. 7F, GFP antibody-based staining of tumor xenografts exhibited that the significant decrease in the percentage of GFP-positive cells were found in CIK-treated mice compared with untreated controls, and the percentage of GFP-positive cells between two CIK-treated groups became statistically significant, suggesting a general downward trend in the number of putative CSCs within tumor xenografts after CIK cell treatment. GFP antibody-based staining method fully validated the quantification of bioluminescence imaging of tumor xenografts. Together, these findings from bioluminescence imaging and GFP antibody-based staining clearly demonstrate that CIK cells treatment leads to the remarkable reduction in the number of putative CSCs within tumor xenografts in NOD/SCID mice, indicating that CIK cells can efficiently kill putative CSCs *in vivo*, which is likely responsible for the significant tumor growth inhibition in mouse xenograft model after CIK cell treatment.

Additionally, H&E staining showed that tumors from animals treated with CIK cells had significantly larger necrotic areas compared to untreated controls (Fig. 7H), and CD5 and CD56 immunohistochemistry assays confirmed the presence of CIK cells infiltrating the tumors formed from CNE2 cells (Fig. 7G).

## DISCUSSION

Our currently study reported the *in vitro* and *in vivo* intense tumor killing activity of CIK cells against putative CSCs of NPC, as fully shown by various efficient *in vitro* and *in vivo* approaches described above. Additionally, recent studies reported the preclinical antitumor activity of patient-derived CIK cells against putative sCSCs of autologous metastatic melanoma [22], and autologous metastatic bone sarcoma and soft-tissue sarcomas [23], as only revealed by *in vitro* little evidences (i.e., visualizing putative CSCs within *in vitro* cultured cancer cells by Oct4 promoter-GFP fluorescence reporter gene strategy, and tumor killing activity assay of CIK cells towards GFP+ putative CSCs), which is still required to be confirmed by the above-mentioned methods (i.e., percentage analysis of SP cells, tumor sphere formation, time-lapse imaging, and *in vivo* optically imaging and quantifying putative CSCs in tumor xenograft-bearing mice, etc). Hence, these preliminary findings from our study and other investigators [22, 23] illustrate the intense tumor killing activity of CIK cells against putative CSCs of NPC, melanoma, and bone sarcoma and soft-tissue sarcomas. However, intensive research work will still be required to be done to explore the intense antitumor killing activity of CIK cells towards putative CSCs of various other cancers.

The strong cytotoxicity of CIK cells against NPC cells, including putative CSCs, prompted us to investigate in detail the mechanisms underlying the tumoricidal effect of CIK cells towards putative CSCs of NPC. The MHC-unrestricted anti-tumor activity of CIK cells is mainly mediated by the interaction of NKG2D with MICA/B and ULBPs[9, 37, 38]. Blocking of NKG2D receptor reduced tumor-killing activity of CIK cells against cancer cells of bone sarcoma and soft-tissue sarcomas [23]. The antitumor activity of CIK cells was equally intense against putative CSCs and non-CSCs of NPC (this study), melanoma [22], and bone sarcoma and soft-tissue sarcomas [23]. In this study, we firstly revealed the direct killing effect of CIK cells against putative CSCs of NPC, at least in part, by NKG2D-ligands recognition.

MICA/B and ULBPs are the main, but not exclusive, ligands recognized by CIK cells; other molecules may be implicated [8]. This could explain the significant reduction but not abrogation of the cytotoxicity of CIK cells observed blocking NKG2D receptor in our study (Fig. 5). A more complete definition of all tumor ligands recognized by CIK cells, their setting of expression and different role in mediating the cytotoxicity of CIK cells may help the identification of subsets of cancer patients that could better benefit from CIK cell-based immunotherapy approaches, suggesting the potential clinical relevance deserving dedicated investigations.

In addition, our observations revealed that CIK cells secreted significant amounts of IFN-γ and IL-6, and slight amounts of IL-2, IL-4, IL-10 and TNF-α, indicating that IFN-γ and IL-6 might play an critical role in anti-tumor activity of CIK cells against tumor cells, which remains to be fully characterized.

Cumulating evidence has revealed that CSCs are responsible for tumorigenicity, invasion, metastasis, therapeutic resistance and tumor recurrence [42, 43]. Thus, the development of methods for *in vitro* optical imaging and non-invasive *in vivo* detection of putative CSCs is of great importance [28, 29]. The commonly used optical molecular imaging techniques include fluorescence imaging and bioluminescence imaging [28–30] which have their own advantages.

At present, the promoter-fluorescence reporter gene strategy has been employed to visualize putative CSCs within *in vitro* cultured cancer cells of hepatocellular carcinoma [35], melanoma [22], and bone and soft-tissue sarcomas [23]. In the aforementioned promoter-reporter gene assay, cancer cells harboring GFP gene under control of a stem cell-specific Oct4 [22, 23] or Nanog promoter [35] were generated to realize visual tracking putative CSCs within a population of *in vitro* cultured cancer cells by *in vitro* fluorescence imaging.

Compared with *in vivo* fluorescence imaging, *in vivo* bioluminescence imaging possesses high spatial resolution and sensitivity and high tissue-penetration depths, and has frequently been used to optically and non-invasively monitor tumor growth, regression and metastasis [28–30], suggesting that *in vivo* bioluminescence imaging should provide the greatest advantage at *in vivo* optically imaging, tracking and quantifying small numbers of cells (i.e., putative CSCs) in human tumor-bearing mice. It is very clear that multimodality imaging approaches can minimize the potential drawbacks of using each imaging modality alone and a tailored combination of two or more imaging techniques may be the best approach for a given experiment [28–30]. Therefore, we firstly developed a new methodology to *in vitro* and *in vivo* visualize putative CSCs based on a lentiviral “CSC detector” vector encoding the copGFP and Luc proteins controlled by human Nanog promoter, which has never been reported previously. Our data from various experimental methods exhibited that GFP+ cancer cells harboring P_Nanog_-GFP-T2A-Luc transgene exhibit the characteristics of CSCs. In this study, GFP+ cancer cells (i.e., putative CSCs) were sorted from NPC cells carrying P_Nanog_-GFP-T2A-Luc transgene to be further used in various experiement mentioned above. By time-lapse video recording, the newly established NPC cell lines harboring P_Nanog_-GFP-T2A-Luc transgene allows us to *in vitro* visualize the whole killing process of CIK cells against GFP+ putative CSCs (Fig. 4), which has never been reported previously. More importantly, the noninvasive *in vivo* bioluminescence imaging allowed us to readily and optically monitor, for the first time, the remarkable reduction in the number of putative CSCs within tumor xenografts in NOD/SCID mice after CIK cell treatment, which was correlated well with the significant decrease in the percentage of GFP-positive cells detected by GFP antibody-based staining method. Therefore, the NPC cell line harboring double reporter genes (i.e., GFP and Luc) under control of a stem cell-specific Nanog promoter allows us to *in vitro* visualize putative CSCs and to *in vivo* optically image and quantify putative CSCs within tumor xenograft-bearing mice, which has never been reported in other cancers.

As mentioned above, real-time imaging of the interactions between CIK cells and NPC cells greatly helps us to understand the killing process of CIK cells against cancer cells, including putative CSCs or tumor spheres. In this study, time-lapse video microscopy fully revealed that when CIK cells were *in vitro* co-cultured with them, each of cancer cells, GFP-labeled or PKH26-labeled putative CSCs, or tumor spheres was usually attacked simultaneously by many CIK cells and finally killed by CIK cells, suggesting that it is quite necessary to achieve sufficient effector to target ratios if cancer cells, including putative CSCs and tumor spheres, are expected to be efficiently killed by CIK cells *in vitro* and *in vivo*. Collectively, our current study firstly revealed the killing whole process of CIK cells against putative CSCs or tumor spheres by time-lapse video microscopy.

As shown above, CSCs/TICs are the root cause for the cancer treatment failure, however, the development of new therapeutic strategies targeting CSCs is currently hindered by the lack of reliable markers for the identification of these CSCs [11–19]. Moreover, one of the key goals in cancer research over the past decade has been to develop therapeutic strategies to efficiently and safely eliminate CSC population for curing the cancer with no or minimal damage to the normal tissues, but a major hurdle to this goal lies in the identification of the key mechanisms that distinguish CSCs from the normal endogenous tissue stem cells [11–19]. One of the major advantages of most immunotherapeutic strategies (e.g., CIK cell-based anticancer immunotherapy) is relatively low or acceptable toxicity against the normal tissues and cells, in contrast to traditional chemotherapy and radiotherapy, whereas the findings from this study and other investigators [8,20,22,23,44] showed that CIK cell-based immunotherapy for cancer exhibited a high cytotoxic activity against cancer cells, including CSCs. Therefore, CIK cell-based therapeutic strategies against CSCs presents a promising and safely approach for cancer treatment.

Although CIK cell therapy for cancer patients exhibits the attractive advantages over other adoptive immunotherapies, evidence supporting the need for therapy-enhancing strategies has come from clinical trials conducted using CIK cell therapy alone. Several strategies have been adopted to enhance CIK cell function and improve the anti-tumor efficacy of CIK cells [45]. These strategies include the use of chimeric antigen receptors (CARs) [46–51] and bispecific antibodies (BsAb) [52–56] to re-direct CIK cells toward specific cancer targets. CAR based immunotherapy has been under development for the last 25 years and is now a promising new treatment modality in the field of cancer immunotherapy and clinical studies of this approach have shown a promising anti-tumor activity [5]. CAR-engineered CIK cells resulted in more efficient tumour cell lysis [8, 46–51]. Moreover, EGFRvIII CAR-engineered T cells exhibited a strong antitumor activity against glioma stem cells expressing mutant EGFRvIII, but not wild-type EGFR [57], while it is necessary to explore whether CAR-engineered CIK cells more efficiently killed stem-like cancer cells of various cancers, compared with CIK cells alone.

The advantages of CIK cell therapy over other types of autologous T cell therapies including CAR T cells are the effective tumor homing abilities and recognition of stress ligands, not tumor-specific antigens, for tumor cell killing [8]. These extend the therapeutic value to numerous types of cancer including solid tumors. Applying CARs to CIK cells improves specific tumor killing, but will still not overcome the effector to target ratio problem [8], indicating that there is an urgent need for the approaches to achieving sufficient effector to target ratios.

The previous studies revealed that CIK cell delivery of the vaccinia virus directly to the tumor can help overcome the localized tumor immunosuppressive environment and increase subsequent immune cell infiltrates to achieve sufficient effector to target ratios [58, 59], suggesting that CAR-engineered CIK cell delivery of the oncolytic virus directly to the tumor may attain sufficient effector to target ratios, which remains to be confirmed. This strategy considers the tumor homing and recognition capabilities of CIK cells along with the constraint of achieving sufficient effector to target ratios.

Other alternatives include co-culturing CIK cells with dendritic cells (DC) to improve activation, enhancement with coadministration of synergistic drugs and cytokines, but these methods increased the complexity of enrichment without providing a dramatic increase in anti-tumor efficacy [8].

In summary, our *in vitro* and *in vivo* findings here demonstrate the intense tumor killing activity of CIK cells against putative CSCs of NPC, indicating that immune targeting of CSCs presents a promising approach for safe cancer treatment. These data prompt us to further investigate the antitumor activity of NPC patient-derived CIK cells against autologous NPC cells, including putative CSCs. On the other hand, as immunotherapy often needs to be accompanied by other therapeutic strategies (e.g., chemotherapy and/or radiotherapy), we will carry out further preclinical and clinical investigations on the prospective potential of targeting putative CSCs of NPC with CIK cells in synergism with traditional treatment strategies such as radiotherapy. CIK cell-based anticancer immunotherapy is becoming a fascinating tool in the fight against cancer, and its further development in the near future is guaranteed. CIK cell-based anticancer immunotherapy could be a very promising adjunct to traditional cancer treatments.

## MATERIALS AND METHODS

### Cell lines and cell culture

Human NPC cell lines (i.e., CNE2 and SUNE1 cells) were obtained from Prof. Qiao Tao (Chinese University of Hong Kong, Hong Kong, China). These NPC cell lines were cultured in RPMI 1640 medium supplemented with 10% fetal bovine serum (FBS) in a humidified incubator with 5% CO_2_ at 37°C. HEK293T cells were maintained in DMEM medium supplemented with 10% fetal bovine serum (FBS; Biological Industries), 1 mM glutamine and 1% nonessential amino acids in a humidified incubator with 5% CO_2_ at 37°C.

### *Ex vivo* generation, expansion and phenotype analysis of CIK cells

Human peripheral blood samples were obtained from healthy volunteer blood donors. All individuals provided their informed consent. Human peripheral blood mononuclear cells (PBMCs) were separated from heparinized peripheral blood by Ficoll–Hypaque density gradient centrifugation, and then washed twice with PBS. Next, PBMCs were re-suspended at 1 × 10^6^ cells/mL in RPMI 1640 (Corning) containing 10% FBS, and cultured in the presence of anti-CD3 antibody (500 ng/mL, Miltenyi, Germany), IFN-γ (100 U/mL, Shanghai Kaimao, China), Polyhydroxyalkanoates (PHA)(10 μg/mL, Huizhou Hongyu, China) and recombinant human interleukin-2 (IL-2)(1000 U/mL, Beijing Sihuan, China) in culture flask for two days. Following this, Fresh medium containing IL-2 and anti-CD3 antibody was replenished every two or three days during culture. Cells were expanded over 3 weeks of time period. Phenotypes of CIK cells were weekly analyzed with a FACSCalibur flow cytometry (BD Biosciences). The following monoclonal antibodies (mAb) were used: CD3-FITC, CD4-FITC, CD8-PE, CD56-APC and CD314-APC (anti-NKG2D) (Miltenyi, Germany).

### CCK8 assay and colony formation assay

Tumor-killing activity of CIK cells against NPC cells was assessed by CCK8 assay. Briefly, CNE2 and SUNE1 cells were plated in 96-well plates at 5 × 10^3^ cells/well, and then CIK cells were added to 96-well plates at specified effector: target ratios (5:1, 10:1, 30:1 and 50:1) in a final volume of 200 μL and co-cultured with NPC cells for 6 h. Tumor cells alone and CIK cells alone were used as the target cells alone group (blank control group) and the effector cells alone group, respectively. The inhibition rate was calculated according to the following formula: inhibition rate (100%) = [1- (A value in experimental well - A value in effector cell wells)/A value in target cell wells] × 100%.

After NPC cells were co-cultured with CIK cells at specified effector: target ratios (5:1, 10:1, 30:1 and 50:1) for 24 h, NPC cells were washed three or four times with PBS to fully remove the suspended CIK cells, and then used for colony formation assay. Colony formation assay was previously fully described [36].

### Transwell migration assay and Boyden invasion assay

After NPC cells were co-cultured with CIK cells at specified effector:target ratio (10:1) for 24 h, NPC cells were washed three or four times with PBS to fully remove the suspended CIK cells, and subsequently collected and counted for transwell migration assay and Boyden invasion assay, as described previously [60].

### Percentages of side population cells (SP cells) analyzed by flow cytometry

CNE2 and SUNE1 cells were firstly co-cultured with CIK cells for 24 h, and then NPC cells were washed three or four times with PBS to remove the suspended CIK cells. Next, NPC cells treated with CIK cells were digested with 0.25% trypsin, washed twice with calcium/magnesium-free PBS, resuspended in ice-cold RPMI-1640 medium (supplemented with 2% FBS) at a concentration of 1 × 10^6^ cells/mL, and incubated at 37°C in a 5% CO_2_ incubator for 90 min. Following this, the changes in the percentage of SP cells were analyzed by flow cytometry (BD FACSAria), as previously fully described [25].

### Tumor spheroid formation assay

After NPC cells were co-cultured for 24 h with CIK cells at different effector: target ratios, NPC cells were washed three or four times with PBS to fully remove the suspended CIK cells, and subsequently cancer cells were collected and counted for tumor spheroid formation assay. Next, 9000 cells were plated in 6-well ultralow attachment plates (Corning, Corning, NY) in serum-free DMEM-F12 (Hyclone), supplemented with 20 ng/mL epidermal growth factor (Peprotech), 10 ng/mL basic fibroblast growth factor (Peprotech), and B27 supplement (1:50 dilution; BD). After 7 days of culture, the number of tumor spheres was counted under an inverted microscope.

### Plasmids and lentivirus vector construction

The plasmid of pL-SIN-P_Nanog_-EGFP, carrying human Nanog promoter, was obtained from Addgene (plasmid 21321). The lentivirus vector of pOct4CR4-pGreenFire1™ EF1-Puro (SR20070-PA-P) was purchased from System Biosciences (SBI). The lentiviral packaging plasmids psPAX2 and pMD2.G were kindly provided by Prof. Didier Trono (University of Geneva, Geneva, Switzerland).

Human Nanog promoter fragment were amplified from pL-SIN-P_Nanog_-EGFP, and then directly inserted into *Eco*R I and *Bam*H I sites of pOct4CR4-pGreenFire1™ EF1-Puro to generate pLV-P_Nanog_-GFP-T2A-Luc. This complete sequence of human Nanog promoter fragment was confirmed through DNA sequencing. Thus, the resultant plasmid of pLV-P_Nanog_-GFP-T2A-Luc harbors both the reporter genes [i.e., GFP and luciferase (Luc)] under control of Nanog promoter and puromycin resistance gene under control of EF-1α promoter.

### Lentivirus production and transduction

To generate stable cell lines, recombinant lentiviruses (named as LV-P_Nanog_-GFP-T2A-Luc) were generated as previously described [61], and subsequently used to infect CNE2 and SUNE1 cells. Next, stable infected cells were selected with puromycin in 4 μg/ml concentration to establish puromycin-resistant stable reporter cell lines (i.e., CNE2 and SUNE1 cells) carrying P_Nanog_-GFP-T2A-Luc transgene, followed by GFP assay via inverted fluorescence microscope (Nikon, Japan) and flow cytomety (BD FACSAria). Moreover, GFP+ and GFP- fractions were sorted for subsequent experiments by fluorescence-activated cell sorting (FACS) (BD FACSAria).

### Western blot analysis

Protein lysates were separated by sodium dodecyl sulfate polyacrylamide gel electrophoresis (SDS-PAGE), and transferred to a polyvinylidene difluoride (PVDF) membrane. The blots were probed with the indicated primary antibodies, followed by HRP (horseradish peroxidase)-labeled secondary antibodies. The hybridization signal was detected using enhanced chemiluminescence (ECL) (Cat.No:KGP1122, KeyGEN BioTECH). GAPDH was used as a loading control. The antibodies used in this study were shown in Table S1.

### PKH26 labeling of cancer cells and sorting of cell populations

CNE2 and SUNE1 cells were labeled using PKH26 Red Fluorescent Cell Linker Kit (Sigma-Aldrich) before cell culture, as described previously [36]. Labeled CNE2 and SUNE1 cells were cultured for about 3–4 weeks. PKH26+ and PKH26- fractions were sorted for subsequent tumor-killing activity assay and time-lapse imaging by fluorescence-activated cell sorting (FACS) (BD FACSAria).

### PKH26 and CM-Dil labeling of CIK cells

In some experiments of this study, to distinguish the CIK cells from GFP-labeled tumor cells, red fluorescent dyes, including PKH26 (Sigma-Aldrich) and CM-Dil (Sigma-Aldrich), were used to label CIK cells, according to the instructions provided by the manufacturer.

### Time-lapse imaging

To observe the interactions between cancer cells or tumor spheres and CIK cells, time-lapse recording was performed using an in-incubator microscope (LumaScope model 600, Etaluma, USA). Image acquisition was begun immediately after CIK cells were added into culture dish. Images were captured every 2 min for 12 h (cells) or 4 min for 24 h (tumor spheres). These images were processed using the LumaView 600 software (Etaluma, USA).

### Anti-NKG2D antibody blocking assay

To investigate the mechanisms underlying the tumoricidal effect of CIK cells, CIK cells were firstly pre-incubated for 30 minutes with 20 μg/mL of inhibitory anti-NKG2D neutralizing antibody (Clone #552866, BD Pharmingen), and subsequently co-cultured with NPC cells for 24 hours, followed by SP cell percentage analysis, colony formation assay and tumor spheroid assay.

### TUNEL staining

After CNE2 and SUNE1 cells were treated with CIK cells for 24 hours, NPC cells were washed three or four times with PBS to fully remove suspended CIK cells, dried at room temperature, fixed with 4% formalin for 15 minutes, and then underwent TUNEL staining by using the TUNEL Apoptosis Detection Kit (KeyGEN BioTECH) according to the manufacturer's instruction.

### Cytokine detection by ELISA

After NPC cells were treated with CIK cells for 24 hours, cell culture supernatants were collected by centrifuging at 2000 × g for 10 min at 4°C and stored at −80°C before use. The cytokine levels in the collected co-culture supernatants were determined by ELISA kit (for IL-2, IL-4, IL-6, IL-10, TNF-α and IFN-γ) (MultiSciences, China) according to the manufacturer's instructions.

### Xenograft experiments in NOD/SCID mice

The animal experiments were carried out in strict accordance with the recommendations in the Guide for the Care and Use of Laboratory Animals of the Southern Medical University. The animal protocol was approved by the Committee on Ethics of Animal Experiments of the Southern Medical University. NOD/SCID mice were purchased from the Model Animal Research Center of Nanjing University, and housed in microisolator cages under aseptic conditions. CNE2 cells (1 × 10^6^ cells) harboring P_Nanog_-GFP-T2A-Luc transgene were resuspended in a mix of PBS and BD Matrigel (BD Biosciences) (1:1), and then subcutaneously injected into the right or left dorsal thigh of mice. 1 week after CNE2 cell implantation, the mice were treated with 1 × 10^7^ CIK cells, 3 × 10^7^ CIK cells or PBS as the untreated control via tail vein injection once every two days. Tumor growth was determined by caliper measurement or *in vivo* bioluminescence (BLI) (see below). Tumor volumes were calculated as previously described [62]. On day 19 after cancer cell implantation, mice were sacrificed, and tumors were dissected, weighed and fixed overnight in 4% paraformaldehyde, dehydrated, paraffin-embedded, sectioned. All surgery was performed under sodium pentobarbital anesthesia, and all efforts were made to minimize suffering of animals.

### *In vivo* bioluminescence imaging

The protocols for whole-animal bioluminescence imaging to noninvasively detect the activity of Luc by the Xenogen IVIS Lumina II Imaging System (Xenogen Corp., Alameda, CA, USA) were previously well described [63, 64].

### Histological and immunohistological examinations

For histology analysis, tumor tissues were fixed with 4% paraformaldehyde (PFA) in PBS, embedded in paraffin, cut into 5 μm thick sections, and then deparaffinized, followed by hematoxylin and eosin staining (H&E staining) according to standard procedures. After deparaffinization and rehydration, the paraffin-embedded sections were subjected to high pressure for 2 min for antigenic retrieval. The slides were incubated overnight at 4°C with the indicated primary antibodies (Table S2).

### Statistical analysis

Data were presented as mean ± SD. Statistical analysis was performed using a SPSS 13.0 software package and Graphpad 5.0 software. Independent-Sample T test was used for comparisons of 2 independent groups. The One-Way ANOVA was used for compare comparisons of multiple groups. The mixed model analysis of variance (Two-Way ANOVA) was employed to assess CIK cytotoxic activity curves *in vivo*. Statistical significance was assessed by the Student's *t*-test (* or ^#^*P* < 0.05; **or ^##^*P* < 0.01).

## SUPPLEMENTARY DATA FIGURES AND TABLES


