# Supplementary material for: Cytokine-induced killer cells efficiently kill stem-like cancer cells of nasopharyngeal carcinoma via the NKG2D-ligands recognition
Source: Oncotarget. 2015 Sep 16;6(33):35023–39. doi: 10.18632/oncotarget.5280 (PMC4741506; doi:10.18632/oncotarget.5280)
Supplement: Supplementary file 1 [file oncotarget-06-35023-s001.pdf]

SUPPLEMENTARY DATA

Expansion and phenotype of CIK cells

$CD3^+/CD56^+$ cells are negligible in fresh human PBMCs, however, they will markedly expand and be generated from T cell precursors. In this study, CIK cells were expanded from fresh PBMCs with the timed addition of IFN-γ, immobilized anti-CD3 antibodies and IL-2, and the absolute number of immune cells increased by more than several hundred-fold for 14 days. Moreover, fluorescence-activated cell sorting (FACS) analyses were employed to characterize the populations of expanded CIK cells. The populations of expanded CIK cells were composed of 3.8% $CD3^+$ $CD56^+$, 64.2% $CD8^+$, 29.6% $CD4^+$, 55.1% $CD3^+$ $CD8^+$ and 49.7% $CD3^+$ $NKG2D^+$ at day 7 after culture, 43.4% $CD3^+CD56^+$, 80.3% $CD8^+$, 10.5% $CD4^+$, 58% $CD3^+$ $CD8^+$ and 55.9% $CD3^+$ $NKG2D^+$at day 14 after culture, and 69.7% $CD3^+CD56^+$, 96.5% $CD8^+$, 3.8% $CD4^+$, 73.4% $CD3^+$ $CD8^+$ and 77% $CD3^+$ $NKG2D^+$at day 21 after culture (Figure S1A). It is clear that during the CIK cell generation, the population of $CD3^+CD56^+$ cells dramatically increased after 14 culture days and after 21 culture days (Figure S1), respectively. This data was confirmed on several occasions, and found to be statistically significant (Figure S1B). Morphologically, the expanded CIK cells (day 14) were large and granular, and growing in aggregate form, as opposed to single cells (Figure S1C).

In addition, the median membrane expression of the NKG2D receptor, mainly responsible for tumor recognition, on expanded CIK cells was 73.7% (day 7), 92.3% (day 14) and 95% (day 21) (Figure S1).

In vitro tumor-killing activity of CIK cells against NPC cell lines

It is well known that CIK cells have been demonstrated to have strong cytotoxicity in a non-MHC-restricted manner. To examine the anti-tumor activity of CIK cells expanded from fresh PBMCs obtained from healthy volunteer, we firstly evaluated their ability to kill NPC cell lines (i.e., CNE2 and SUNE1 cells) *in vitro* by using CCK8 assay. As expected, fresh PBMCs did not destroy CNE2 and SUNE1 target cells; however, expanded CIK cells markedly broke CNE2 and SUNE1 target cells down in a dose-dependent manner (Figure 1A). As showed in Figure 1A, the average specific tumor killing was 74.1 ± 1.1%, 67.7 ± 1.3%, 59.5 ± 2.7% and 58.4 ± 2.6% (mean ± SD) (for CNE2 cells), and 88.3 ± 2.9%, 81 ± 1.2%, 70.3 ± 2.8% and 62.3 ± 3.3% (mean ± SD) (for SUNE1 cells) at 50:1, 30:1, 10:1 and 5:1 effector/target ratio (E:T ratio), respectively ($n = 6$), indicating that increasing the E:T ratio directly correlated positively with the cytotoxic effect of CIK cells. Colony formation assay demonstrated that CIK-treated CNE2 and SUNE1 target cells demonstrated a gradually decreased colony forming ability with an increase in E:T ratio (Figure 1B). Additionally, CNE2 and SUNE1 target cells treated with CIK cells for 24 h illustrated significantly reduced cell migration and invasion, compared with controls (Figure 1C). Therefore, CIK-treated CNE2 and SUNE1 target cells showed the remarkable decrease in colony forming ability, and motile and invasive ability. Summarily, these data suggest that heterogeneous CIK cell population, which was largely generated from human PBMCs, had a strong cancer cell killing activity (CKA) *in vitro* against NPC cells.

CIK cells inhibited tumor growth of NPC cells in nude mice

Furthermore, we also assessed the antitumor activity of CIK cells in nude mouse xenograft assays (Figure S4). CIK cells were injected intravenously at 1×10^7 cells per mouse, and as a result, significantly inhibited *in vivo* tumor growth by 68% (Figure S4A, S4B; $p < 0.05$). Cisplatin (DDP), used as a reference drug, strongly inhibited the *in vivo* tumor growth (Figure S4A, S4B, S4C). On day 34, all tumors were isolated from nude mice and weighed, which demonstrated the strong anti-tumor effect of CIK cells against CNE2 cells (Figure S4A, S4C). The body weights of tumor-bearing nude mice were examined to evaluate the toxicity. Control and CIK-injected nude mice showed a body weight gain of 121–122%; whereas DDP-treated mice showed a body weight gain of 105%, which shows the typical characteristics of a cytotoxic anti-cancer drug (data not shown).

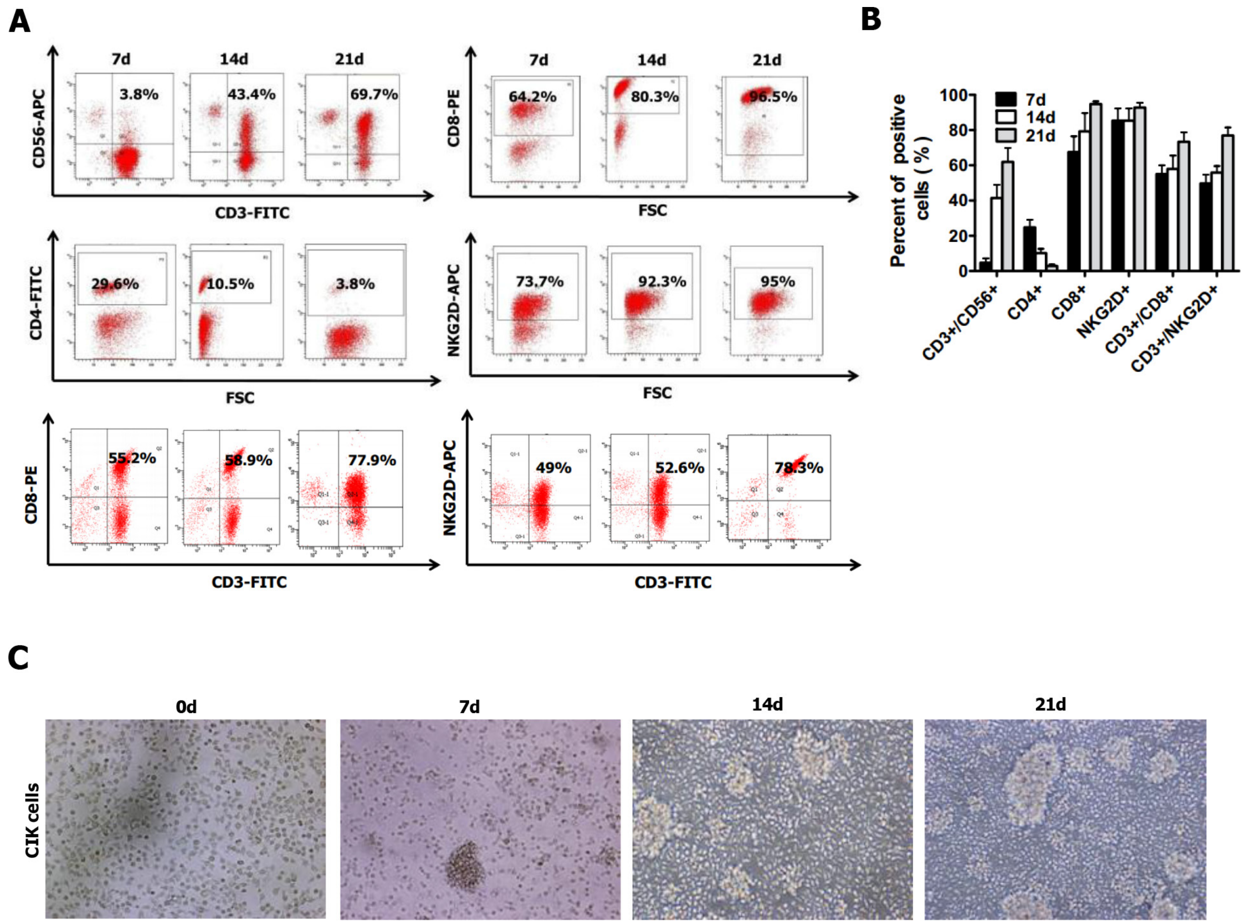

Supplementary Figure S1: The phenotypic characterization of CIK cells by flow cytometry. A. Representative flow cytometry analysis of the surface marker of CIK cells. **B.** The phenotypes of CIK cells. **C.** Representative pictures of the generation and expansion of CIK cells.

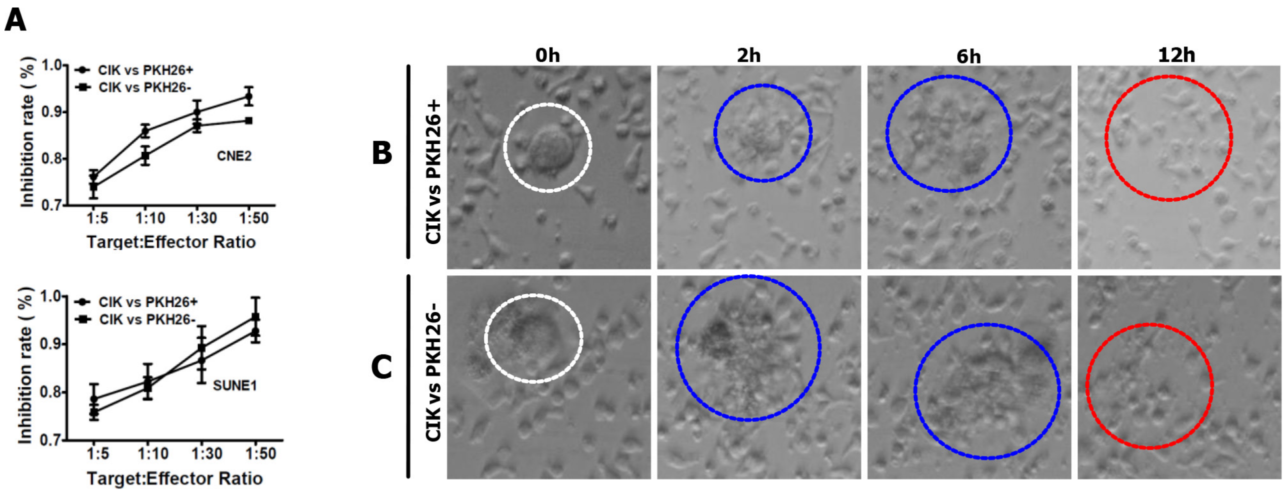

Supplementary Figure S2: CIK cells have direct killing effect on stem-like cancer cells of NPC. A. The antitumor activity of CIK cells was equally intense against PKH26 (+) and PKH26 (-) NPC cells. **B–C.** Time-lapse imaging (see Supplemental Movie 3 and 4) used to observe the interactions between CIK cells and PKH26 (+) cells or PKH26 (−) cells sorted from PKH26-labelled CNE2 cells. Time-lapse images at times 0 h, 2 h, 6 h and 12 h are shown in (B) and (C), respectively. The white dashed circles enclose one PKH26 (+) cell in (B) and one PKH26 (−) cell in (C), respectively. Cancer cells situated in the regions surrounded by these blue dashed circles in (B) and (C) were being scavenged by CIK cells, while cancer cells located in the areas surrounded by these red dashed circles in (B) and (C) had been scavenged by CIK cells.

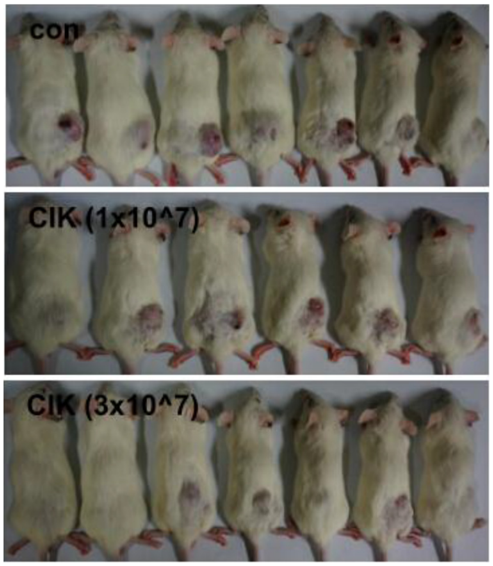

Supplementary Figure S3: CIK cells suppressed tumor growth of NPC cells in NOD/SCID mice. As mentioned in Materials and methods section, 1×10^6 CNE2 cells harboring P_{Nanog}-GFP-T2A-Luc transgene were injected subcutaneously into NOD/SCID mice (*n* = 20). 7 days after tumor cell implantation, 1×10^7 and 3×10^7 CIK cells were infused by tail vein injection into recipient mice. Representative pictures of tumors formed at the end of experiment.

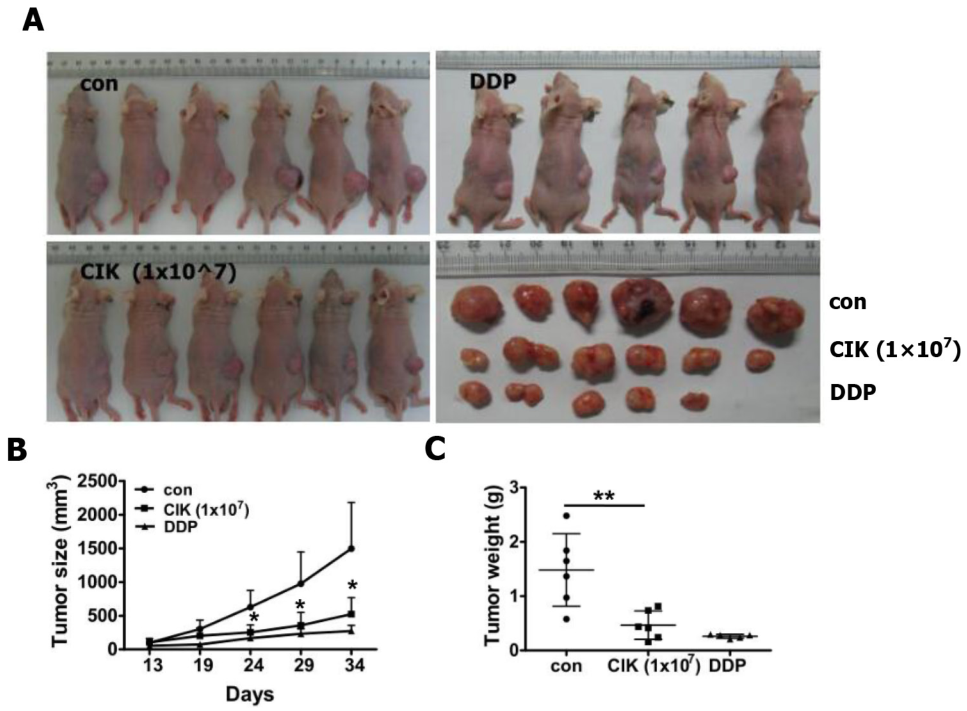

Supplementary Figure S4: CIK cells inhibited tumor growth of NPC cells in nude mice. CNE2 cells were injected subcutaneously into nude mice (n = 17). 7 days after tumor cell implantation, CIK cells were injected intravenously through tail vein into recipient mice (n = 6) once every five days at doses of 1×10^7 cells per mouse. Mice (n = 6) injected with PBS were used as the negative control, while cisplatin (DDP, 80 µg/mouse) intraperitoneally injected into nude mice (n = 5) was used as the reference drug. Statistical significance was determined using the Student's t-test versus PBS-treated control group ($*p < 0.05$, $**p < 0.01$). **A.** Representative picture of tumors formed at the end of experiment. **B.** Growth curve of tumor volumes. Tumor size was estimated by serial calipation. Each data point represents the mean ± SD of 6 mice. **C.** Tumors were weighted. Each data point represents the mean ± SD of 6 mice.

Supplementary Table S1: List of antibodies and suppliers used for Western blot

Antibody	Isotype	Suppliers
E-cadherin	Mouse IgG2a	BD Biosciences
α-catenin	Mouse IgG1	BD Biosciences
Vimentin	Mouse IgG1	BD Biosciences
N-cadherin	Mouse IgG1	BD Biosciences
Klf4	Rabbit IgG	Abcam
Oct4	Rabbit IgG	Abcam
Sox2	Rabbit IgG	Abcam
Nanog	Rabbit IgG	Abcam
GAPDH	Rabbit IgG	Bioss

Supplementary Table S2: List of antibodies and suppliers used for immunohistochemistry

Antibody	Isotype	Suppliers
BrdU	Mouse Ig	GE Healthcare
Ki67	Rabbit IgG	Abcam
GFP	Mouse Ig	Bioworld
CD5	Mouse IgG1	eBioscience
CD56	Mouse Ig	Novus

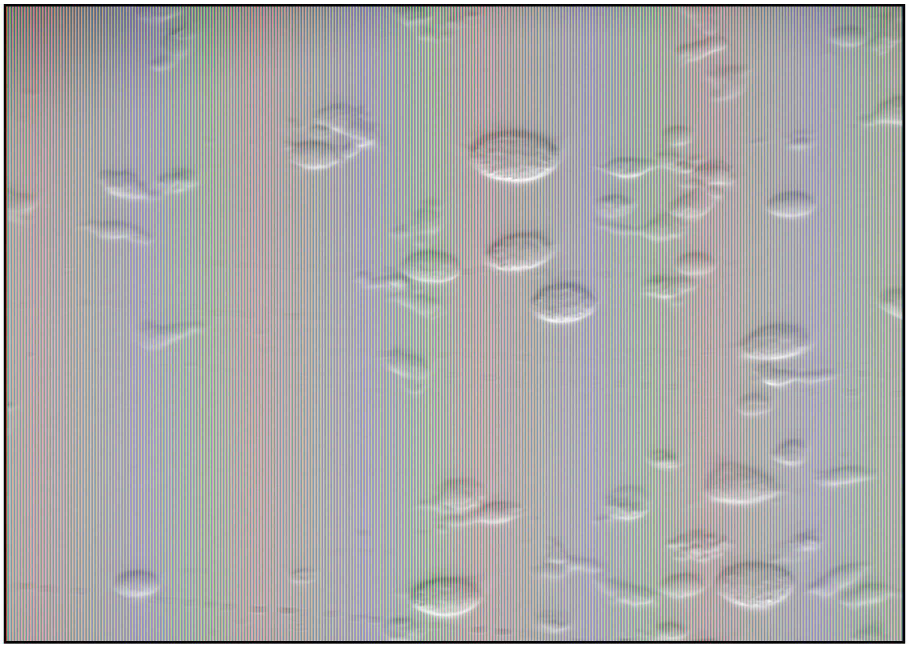

Supplementary Movie S1: Time-lapse imaging used to observe the interactions between CNE2 cells and CIK cells. The video corresponds to the experiment shown in Figure 4B. Representative time-lapse images at times 0 h, 2 h, 6 h and 12 h are shown in Figure 4B. The white dashed circles enclose cancer cells. Cancer cells located in the regions surrounded by these blue dashed circles were being scavenged by CIK cells, while cancer cells situated in the areas surrounded by red dashed circle had been scavenged by CIK cells.

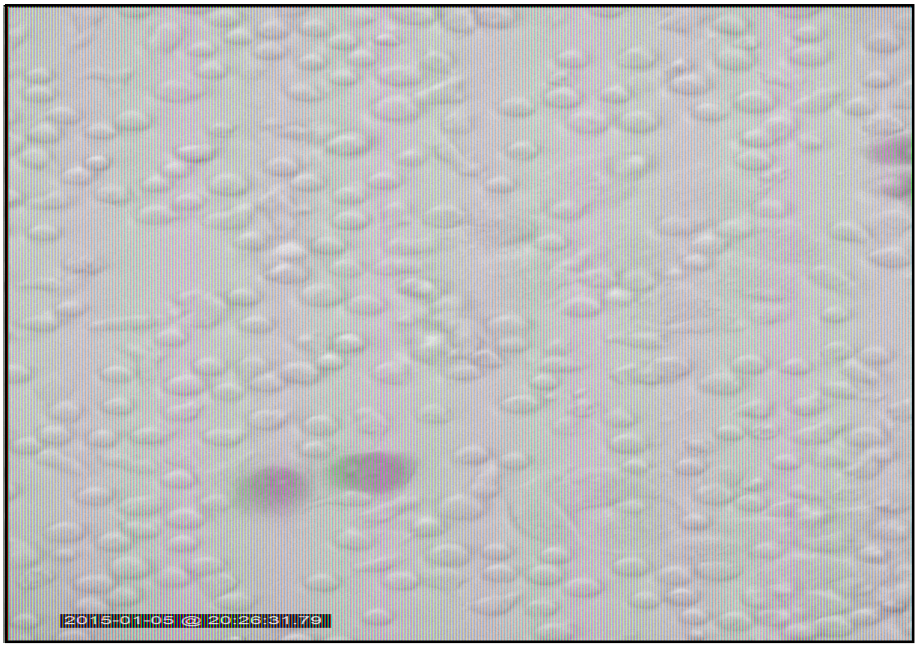

Supplementary Movie S2: Time-lapse imaging used to observe the interactions between CIK cells and CNE2 cells harboring P_{Nanog}-GFP-T2A-Luc transgene. The video corresponds to for the experiment shown in Figure 4C. Representative time-lapse images at times 0 h, 2 h, 6 h and 12 h are shown in Figure 4C. The black and white dashed circles enclose two GFP+ and two GFP- cancer cells, respectively. GFP+ and GFP- cancer cells located in the regions surrounded by yellow and blue dashed circles, respectively, were being scavenged by CIK cells, while GFP+ and GFP- cancer cells situated in the areas surrounded by red dashed circle had been scavenged by CIK cells.

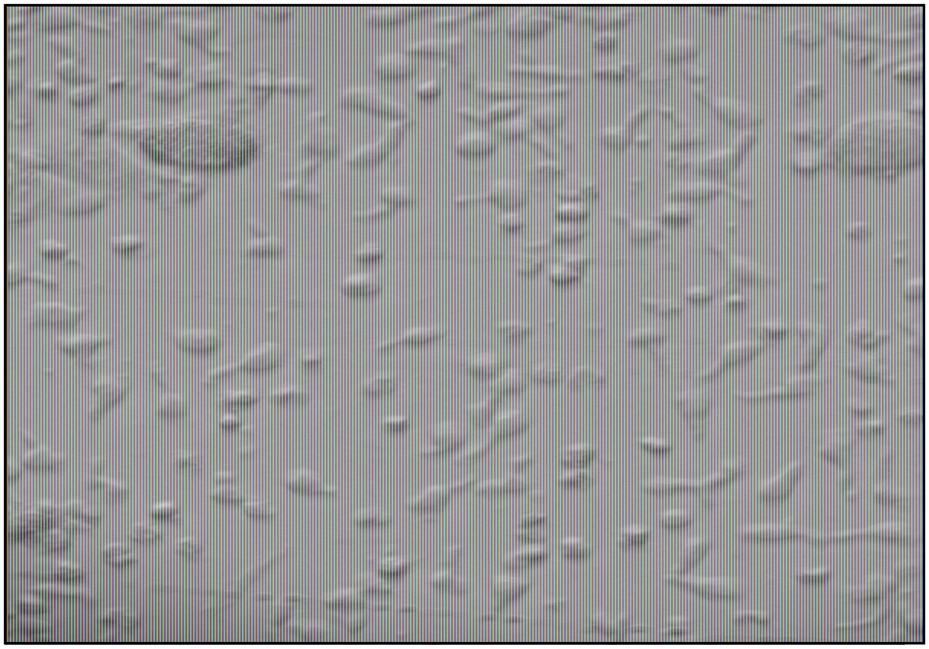

Supplementary Movie S3: Time-lapse imaging used to observe the interactions between CIK cells and PKH26+ cells sorted from PKH26-labelled CNE2 cells. The video corresponds to the experiment shown in Fig. S2B.

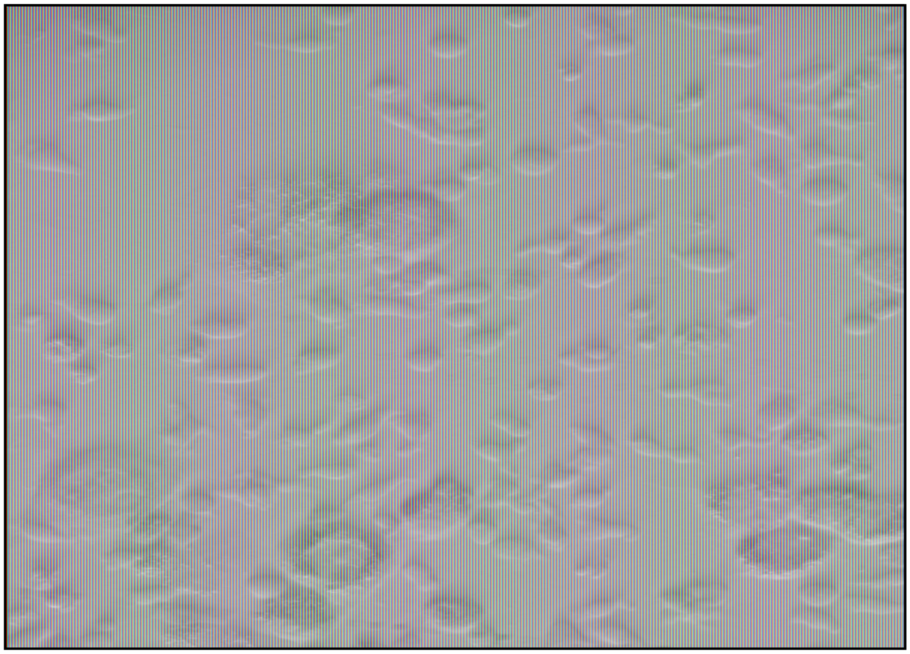

Supplementary Movie S4: Time-lapse imaging used to observe the interactions between CIK cells and PKH26- cells sorted from PKH26-labelled CNE2 cells. The video corresponds to the experiment shown in Fig. S2C.

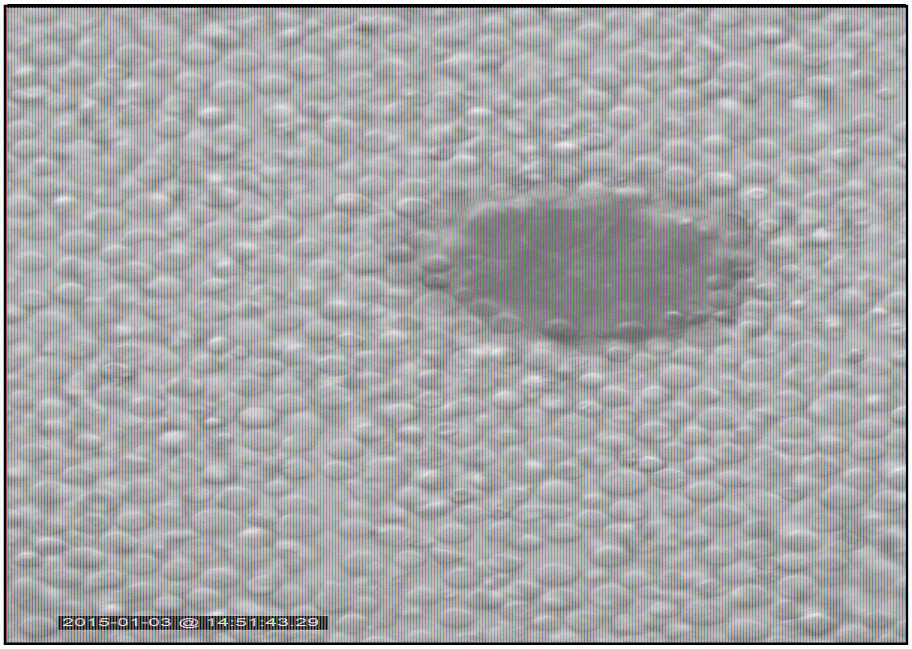

Supplementary Movie S5: Time-lapse imaging used to observe the interactions between GFP+ tumor sphere (derived from GFP+ cells sorted from CNE2 cells harboring P_{Nanog}-GFP-T2A-Luc transgene) and CIK cells. The video corresponds to the experiment shown in Fig. 4E. Representative time-lapse images at times 0 h, 2 h, 6 h and 12 h are shown in Fig. 4E. The black dashed circle encloses GFP+ tumor sphere. GFP+ tumor sphere surrounded by yellow dashed circles was being scavenged by CIK cells, while GFP+ tumor sphere situated in the areas surrounded by red dashed circle had been scavenged by CIK cells.
